# Structurally Tailored Antibacterial Quaternary Ammonium Salts with Ionic Liquid Properties for Antimicrobial Purposes: Design and Thermophysical Insights

**DOI:** 10.1021/acssuschemeng.5c07350

**Published:** 2025-10-27

**Authors:** Paola Marzullo, Salvatore Marullo, Alessandro Presentato, Enrico Tornatore, Carla Rizzo, Rosa Alduina, Michelangelo Gruttadauria, Francesca D’Anna

**Affiliations:** † Department STEBICEF, University of Palermo, Viale delle Scienze, Ed. 17, Palermo, Italy; ‡ Sustainable Mobility Center (Centro Nazionale per la Mobilità Sostenibile—CNMS), Via Durando 39, 20158 Milano, Italy

**Keywords:** quaternary ammonium salts (QASs), ionic liquids (ILs), structure−activity relationship, antibacterial properties, *Pseudomonas delhiensis*, thermophysical characterization

## Abstract

Biofilm-forming bacteria pose therapeutic and industrial challenges due to their heightened resistance to antimicrobials and their role in surface contamination and material degradation. Quaternary ammonium salts (QASs), especially those with ionic liquid (IL)-like properties, have emerged as promising agents for controlling biofilms. This study reports the synthesis and characterization of a series of structurally tailored antibacterial QAS, some displaying IL-like behavior, and evaluates their antimicrobial activity. The tested organism was *Pseudomonas delhiensis* PS27, a multidrug-resistant Gram-negative environmental strain isolated from a site contaminated with perfluoroalkyl and polyfluoroalkyl substances (PFASs). Owing to its high resilience, *P. delhiensis* PS27 serves as a robust model for assessing the efficacy of biocidal agents. Thermophysical properties, including phase transitions and thermal stability, were evaluated using differential scanning calorimetry (DSC) and thermogravimetric analysis (TGA) to ensure the compounds’ practical applicability. Among the most effective compounds, the ionic liquid [C_14_C_2_OHMor]Br and the piperidinium salt [C_1_C_14_Pip]Br exhibited notable antibacterial activity, though the morpholinium derivative showed reduced efficacy likely due to oxygen-related lower toxicity. The [C_1_C_14_Pip]_3_[Trim] demonstrated ionic liquid properties combined with strong antibacterial effects and high thermal stability. Additionally, novel di-imidazolium ILs, [*o*-xyl­(C_8_Im)_2_]­[Docu]_2_ and [*o*-xyl­(C_8_Im)_2_]­[Tos]_2_, showed enhanced antimicrobial performance. Overall, the study highlights key structure–activity relationships and identifies promising candidates for the development of advanced antifouling technologies, particularly polymer-based coatings and slippery-liquid-infused porous surfaces (SLIPs).

## Graphical abstract

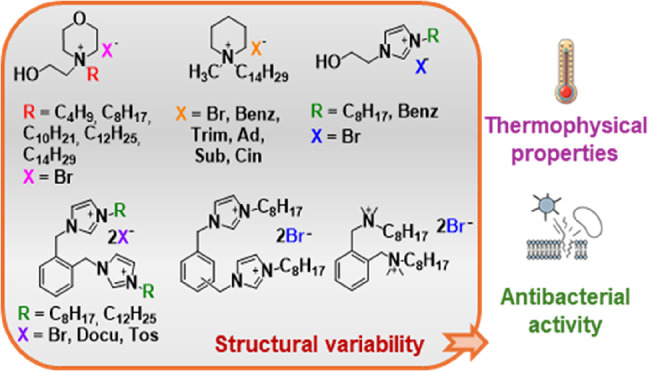

## Introduction

Bacterial species can form highly organized, surface-associated communities known as biofilms. A key aspect of the biofilm formation process relies on the production of an extracellular polymeric substance (EPS) that encapsulates and stabilizes the biofilm structure. Within this protective environment, bacterial cells highlight enhanced resistance to antimicrobial agents and increased tolerance to environmental stresses.^1,2^

In aquaculture systems, bacterial strains with a high proficiency to form biofilms play a pivotal role in biofouling, adversely impacting infrastructures, and resulting in considerable economic losses.^3,4^

Accordingly, the early inhibition of biofilm formation, specifically by preventing planktonic cells from irreversibly adhering to surfaces through the application of antifouling agents, is of paramount importance. This strategy is especially crucial for *Pseudomonas* spp., renowned for their opportunistic nature, robust biofilm-forming capacity, and prominent contribution to the deterioration of aquatic environments.^3,5,6^

A recent study provided an overview of the existing literature on antifouling materials based on quaternary ammonium salts, emphasizing their wide range of applications.^7^ Furthermore, a dedicated review was conducted to highlight the potential of these systems for use in marine environments and water treatment technologies, reflecting continued interest in this area of research.^8^

Quaternary ammonium salts (QASs), including compounds with ionic liquid properties, are considered interesting antibiofilm agents.^8–13^

Ionic Liquids (ILs), unlike common salts, thanks to their high thermal stability, nonflammability, low melting point (<100 °C), and negligible vapor pressure, are promising “green alternatives” to organic solvents in diverse areas such as synthesis and catalytic chemistry, for liquid–liquid extraction or electrochemical processes.^14–22^

ILs possess an organic cation based on alkyl-substituted nitrogen or phosphorus (imidazolium, ammonium, pyridinium, pyrrolidinium, morpholinium, piperidinium or phosphonium) and anions of inorganic or organic type, with different water solubility, such as bromide (Br^–^), chloride (Cl^–^), hexafluorophosphate (PF_6_^–^), bis­(trifluoromethane)­sulfonilimide ((CF_3_SO_2_)_2_N^–^), tetrafluoroborate (BF_4_^–^), dicyanamide ((CN)_2_N^–^), alkyl sulfate (RSO_4_-), carboxylate (RCOO^–^), and also amminoacid.^23^

Antimicrobial ILs and QASs share the mechanism of action. They are known as “membrane-active biocides” because their cationic and amphiphilic properties allow bacterial membrane destabilization, increased permeability, membrane leakage, and cellular death. In particular, the cation and the alkyl side chain interact with the cellular surface through electrostatic and hydrophobic interactions.^24,25^ As reported in several works, antibacterial activity increases with chain length to a cutoff point. When a critical length of the alkyl chain is reached, molecules closely resemble the natural components of the bilayer, with consequent limited cell damage. In addition, poor solubility and possible micellization may limit diffusion into the membrane.^26,27^ QASs are also known to interact with DNA and to interfere with the function of proteins with consequent metabolic dysregulation and oxidative stress.^27^

Despite the wide range of applications of QASs and ILs, their ecotoxicology and environmental fate are of particular concern.^28^ The known stability of these compounds may result in their persistence as contaminants in air, water, and soil. Moreover, structural features that promote antibacterial activity result in poor biodegradability and, generally, increased toxicity even to eukaryotic cells.^29–31^

To address this problem and the growing incidence of QASs resistance, researchers have explored a vast range of cation–anion combinations, estimated to be 10^18^, varying the length of the alkyl side chain of the cation and its functionality to obtain structurally optimized ILs and QASs.^9,10,32,33^ Indeed, ILs are often considered ″designer″ or ″task-specific″ solvents because of the great potential for structural modification to achieve specific properties and applications.

Considering previous structure–activity correlation studies and responding to the urgent need for new antimicrobial agents, the present study focuses on the synthesis of QASs shown in Scheme 1, some of which have ionic liquid properties.

**Scheme 1:**
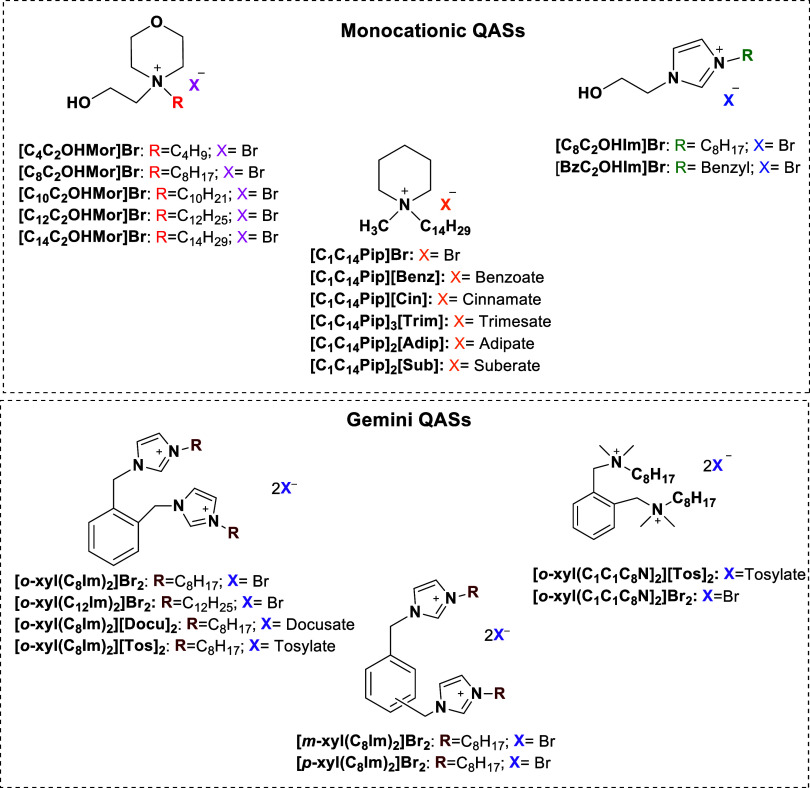
Synthesised Salts Tested for Their Antibacterial Activity

To describe them, an acronym with the general formula **[ABC]**_**x**_ **[D]**_**y**_ was adopted, where [ABC] is the cation and [D] is the anion; x and y are numbers indicating the stoichiometric ratio cation/anion. A and B are substituents at the nitrogen atom, C can be replaced by the abbreviation according to the type of cyclic cation as Pip, Mor, Im for Piperidinium, Morpholinium and Imidazolium, respectively. In cyclic salts, the alkyl chains at the nitrogen, represented by the letters A and B in the general formula, are indicated by C*_n_*, where *n* is the number of carbon atoms. The hydroxyethyl side chain is represented by C_2_OH. For example, the formula [C_4_C_2_OHMor]­Br is for *N*-butil-*N*-hydroxyethyl-morpholinium bromide salt. In the case of alkyl ammonium salts, the letter **C** in the general formula is replaced by the members C*_n_*, which represent two other alkyl chains.

This work reports, also, the synthesis and antibacterial evaluation of dicationic salts (Gemini QASs) of aliphatic or aromatic type, with an aromatic spacer. These were nominated **[** ***a*** **-xyl­(AIm)**_**2**_ **]­[D]**_**2**_ or **[** ***a*** **-xyl­(ABCN)**_**2**_ **]­[D]**_**2**_ for imidazolium and alkylammonium salts, respectively. Also, in this case, **D** is substituted with an anion acronym. Two cationic heads on the aromatic spacer can have three different substitution patterns indicated by the letter **a** in the general formula, which can be *o*, *m* or *p*, standing for *ortho*, *meta* or *para* isomers. Again, letters **A**, **B**, and **C** are substituted by C*_n_* corresponding to the alkyl chain at the nitrogen of the head groups, with a corresponding number of carbon atoms.

In detail, to assess the influence of a second cationic center, a series of dicationic salts was prepared. Moreover, the role of aromaticity was studied by synthesizing mono- and dicationic salts bearing either aliphatic or aromatic cations. The incorporation of oxygen atoms and hydroxyethyl chains into morpholinium-based structures, alongside systematic variations in the length of the alkyl side chains, enabled a detailed evaluation of lipophilicity effects. For piperidinium-based salts, the impact of the counterion and different cation-to-anion stoichiometric ratios were examined. Particularly, anions of aromatic (benzoate, cinnamate, and trimesate) and aliphatic (adipate and suberate) nature were considered in the design of salts. Furthermore, to obtain additional information about the influence on antibacterial and thermal properties of different stoichiometries between cation and anion (1:1, 2:1, or 3:1), anions with one (benzoate and cinnamate), two (suberate and adipate) and three (trimesate) charged head groups were selected. Moreover, extensive conjugation in the cinnamate anion and different lengths of alkyl spacers for adipate and suberate anions were analyzed. Also, for Gemini QASs, the nature of the cationic headgroup (aliphatic vs. aromatic), the side chain length, and the anionic counterpart were systematically varied.

Synthesized compounds were characterized in their physicochemical properties using differential scanning calorimetry (DSC) and thermogravimetric analysis (TGA) for phase transition and thermal stability, respectively. Indeed, in view of potential applications, it is important to understand thermophysical properties and the liquid operating temperature range.

The Antimicrobial activity was tested against a Gram-negative bacterium, *Pseudomonas delhiensis* PS27, which was previously isolated from an environmental niche contaminated by hazardous perfluoroalkyl and polyfluoroalkyl chemicals (PFASs). This strain, highly resistant to environmental stressors, has been studied for its capacity to bioaccumulate perfluorohexane sulfonate within the cells under alkanotropic conditions.^34^

The choice of a Gram-negative strain was intentional. Indeed, due to the presence of the outer membrane and lipopolysaccharide layer, lower susceptibility to ILs was envisaged compared to Gram-positive bacteria.^35,36^ To the best of our knowledge, this is the first case in which the antimicrobial activity of a set of QASs and ILs has been tested against a highly resilient *Pseudomonas* strain, revealing critical structure–property relationships.

The structural modifications introduced in this study were found to significantly influence cation–anion interactions, lipophilicity, crystalline architecture, and thermal degradation behavior, with consequential effects on both thermophysical and antibacterial properties. In particular, morpholinium-, piperidinium-, and di-imidazolium- based salts have proven to be promising candidates as next-generation antimicrobial agents. Therefore, this research represents an important contribution to the understanding of structure–property relationships in QASs and ILs, providing a solid rational basis for the design of new compounds effective against highly resilient environmental bacterial strains capable of biofilm formation.

## Experimental Section

### Materials and Methods

All solvents and reagents were obtained from commercial sources and were used without purification. 4-butylmorpholine, 2-bromoethanol, 1-methylpiperidine, 1-bromodecane, 1-bromododecane, 1-bromotetradecane, α,α′-dibromo-*o*-xylene, α,α′-dibromo-*m*-xylene, α,α′-dibromo-*p*-xylene, Benzoic acid, Cinnammic acid, Trimesic acid, Suberic acid, Dioctyl sodium sulfosuccinate and Amberlite IRA-400 ion-exchange resin were purchased from Sigma-Aldrich (Burlington, MA, US).

4-(2-hydroxyethyl)­morpholine, Octylbromide, 1-(2-hydroxyethyl)-imidazole, 1-octyl-imidazole, *N,N*-dimethyloctylamine, *p*-toluenesulfonic acid were purchased from Fluorochem (Hadfield, UK). All solvents (ACS grade) were purchased from Merck (Milan, IT).

^1^H NMR and ^13^C NMR spectra were recorded at room temperature on Bruker (Billerica, MA, US) Advance II 400 MHz (9.4 T) operating at 400 MHz for ^1^H and 101 MHz for ^13^C; DMSO-d_6_ or CDCl_3_ were used as solvents and TMS as an internal standard.

Thermogravimetric analysis (TGA) was performed using a TA Instruments SDT650 (New Castel, DE). Samples, weighing between 5 and 10 mg, were accurately measured and placed in alumina pans. The analysis began with an isothermal step at 100 °C for 20 min to remove any residual moisture. Subsequently, the samples were heated at a rate of 5 °C/min up to 520 °C under a nitrogen atmosphere (flow rate: 50 mL/min). Upon reaching 520 °C, the gas atmosphere was switched to oxygen (O_2_) (flow rate: 50 mL/min), and heating continued up to 900 °C at a rate of 20 °C/min. The obtained data were processed using the instrument’s associated software, allowing the determination of mass loss as a function of temperature and the evaluation of the thermal stability and oxidative behavior of the materials.

Differential scanning calorimetry (DSC) measurements were performed using Differential Scanning Calorimeter (TA Instrument Inc., New Castle, DE) equipped with a refrigerated cooling unit. Samples (∼4 mg) were weighed into hermetically sealed TA Tzero aluminum pans. The analysis was carried out with heating and cooling rates of 10 °C min^–1^ under a nitrogen atmosphere with a flow rate of 50 mL min^–1^. Two heating/cooling cycles were performed over a temperature range of 0 to 190 °C.

### Bacterial Growth Inhibition Assay

The minimal inhibitory concentration (MIC) of quaternary ammonium salts (QASs), including compounds exhibiting ionic liquid (IL) properties and based on morpholinium, piperidinium, imidazolium, dialkylammonium, and di-imidazolium cations, was assessed against the *Pseudomonas delhiensis* PS27 strain. The evaluation was performed by incubating bacterial cells at a final concentration of 0.05% (v/v), corresponding to 5 × 10^5^ colony-forming units (CFU) per milliliter, derived from an overnight culture (approximately 16 h) grown in tryptic soy broth (TSB) at 37 °C with shaking (180 rpm). The TSB medium consisted of the following components (g/L): casein peptone (17), soya peptone (3), sodium chloride (5), dipotassium hydrogen phosphate (2.5), and glucose (2.5).

At the time of inoculation, bacterial cultures (180 μL final volume) were dispensed into flat-bottom 96-well microtiter plates, being the bacterial cells challenged for 24 h with increasing concentrations of the selected biocides. Bacterial growth was monitored by measuring the optical density (OD) at 600 nm using a Synergy HT BioTek microplate reader, thus providing, in an indirect way, the extent of bacterial growth. All measurements were conducted in triplicate, and OD values were corrected by subtracting the absorbance of the culture medium alone. Data are expressed as average OD values ± standard deviation (SD).

### General Procedure for the Synthesis of Bromide Salts Based on 4-Alkyl-4­(2-hydroxyethyl)­morpholinium, 1-Methyl-1-tetradecyl-piperidinium and 1-(2-Hydroxyethyl)-3-alkyl-imidazolium Cations

The quaternization reactions were performed by alkylating 4-(2-hydroxyethyl)­morpholine, 1-methylpiperidine, and 1-(2-hydroxyethyl)-imidazole with the corresponding bromoalkanes in stoichiometric amounts. The reactions were carried out in acetonitrile under reflux at 70 °C for 24 h.

The [C_4_C_2_OHMor]Br salt was synthesized by alkylation of 1-butylmorpholine with an equimolar amount of 2-bromoethanol in acetonitrile at 70 °C for 24 h. The imidazolium-based salt [BzC_8_Im]Br was obtained via alkylation of 1-octyl-imidazole with a stoichiometric quantity of benzyl bromide in isopropanol at 85 °C for 18 h.

After completion of the reactions, the solvents were removed under reduced pressure, and the crude products were purified by precipitation from ethyl acetate or diethyl ether. The resulting salts were dried under vacuum until constant weight.

Synthetic details and NMR characterization of the resulting salts are provided in the Supporting Information file (Page S3–5 and S7)

### General Procedure for the Synthesis of Dicationic Bromide Salts

A solution of 1-octyl-imidazole, *1*-dodecyl-imidazole, or *N,N*-dimethyloctylamine (2.2 equiv) in isopropanol was added dropwise to a stirred solution of *o*-, *m*-, or *p*- α,α′-dibromo-xylene (1 equiv) previously dissolved in isopropanol. The reaction mixture was heated at 85 °C for 24 h under stirring, and the progress of the reaction was monitored by thin-layer chromatography (TLC). After completion, the solvent was removed under reduced pressure, and the crude residue was washed with acetone or diethyl ether to afford the corresponding dicationic bromide salts. Synthetic details and NMR characterization of the resulting salts are provided in the Supporting Information file (Page S8).

### General Procedure for Anion Exchange

The exchange of the bromide anion with tosylate, adipate, suberate, trimesate, cinnamate, and benzoate anions was performed using Amberlite IRA-400 resin in the chloride form, following a previously reported procedure.^37^ The resin was first converted to the hydroxide form by washing with an aqueous sodium hydroxide solution, and then thoroughly rinsed with deionized water until neutral pH was reached.

The bromide salts were dissolved in a methanol/water mixture (70:30, v/v) and passed through the ion-exchange column, using the same solvent mixture as the eluent. The eluate was collected in a flask containing a stoichiometric amount of the desired acid, and elution was continued until the effluent reached neutral pH. The solvent was then removed under reduced pressure, and the resulting residue was washed with diethyl ether to yield the corresponding salts.

The exchange of bromide with the docusate anion was carried out by a metathesis reaction in dichloromethane at room temperature, using a stoichiometric amount of dioctyl sodium sulfosuccinate salt (AOT). The precipitated NaBr byproduct was removed by filtration, and the organic phase was washed with water until no turbidity was observed upon testing the aqueous layer with a silver nitrate (AgNO_3_). Synthetic details and NMR characterization of the resulting salts are provided in the Supporting Information file (Page S5 and S9–10)

## Results and Discussion

### Synthesis of Monocationic and Gemini QASs

The study began with the synthesis of morpholinium-based derivatives with hydroxyethyl and alkyl chains of different lengths. Scheme 2, pathway (a), illustrates the synthesis of the **[C**_**4**_ **C**_**2**_ **OHMor]­Br** salt via the reaction of 4-butylmorpholine with 2-bromoethanol. Morpholinium salts bearing longer alkyl side chains were synthesized through the alkylation of 4-(2-hydroxyethyl)­morpholine using the corresponding alkyl halides, as shown in Scheme 2 pathway (b). As reported in the literature, the longer alkyl chain may increase the antibacterial activity.^38^ Conversely, the morpholinium ring and the hydroxyethyl chain may make the derivatives less toxic to other organisms.^39^

**Scheme 2:**
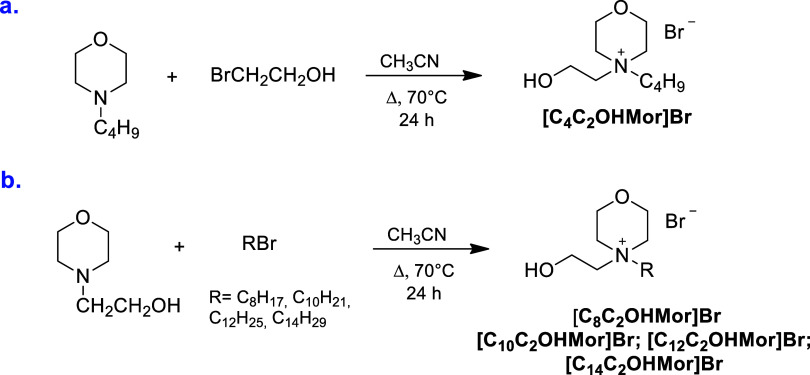
Synthesis of 4-(2-Hydroxyethyl)­morpholinium-Based Salts

The 1-methyl-1-tetradecyl-piperidinium derivative **[C**_**1**_ **C**_**14**_ **Pip]­Br**, obtained from 1-methylpiperidine and 1-bromotetradecane as shown in Scheme 3 – path a, was used in an anion exchange protocol on Amberlite resin IRA-400 (chloride form) to obtain salts of [C_1_C_14_Pip]^+^ bearing different counterions (Scheme 3 - path b and c).^40^

**Scheme 3:**
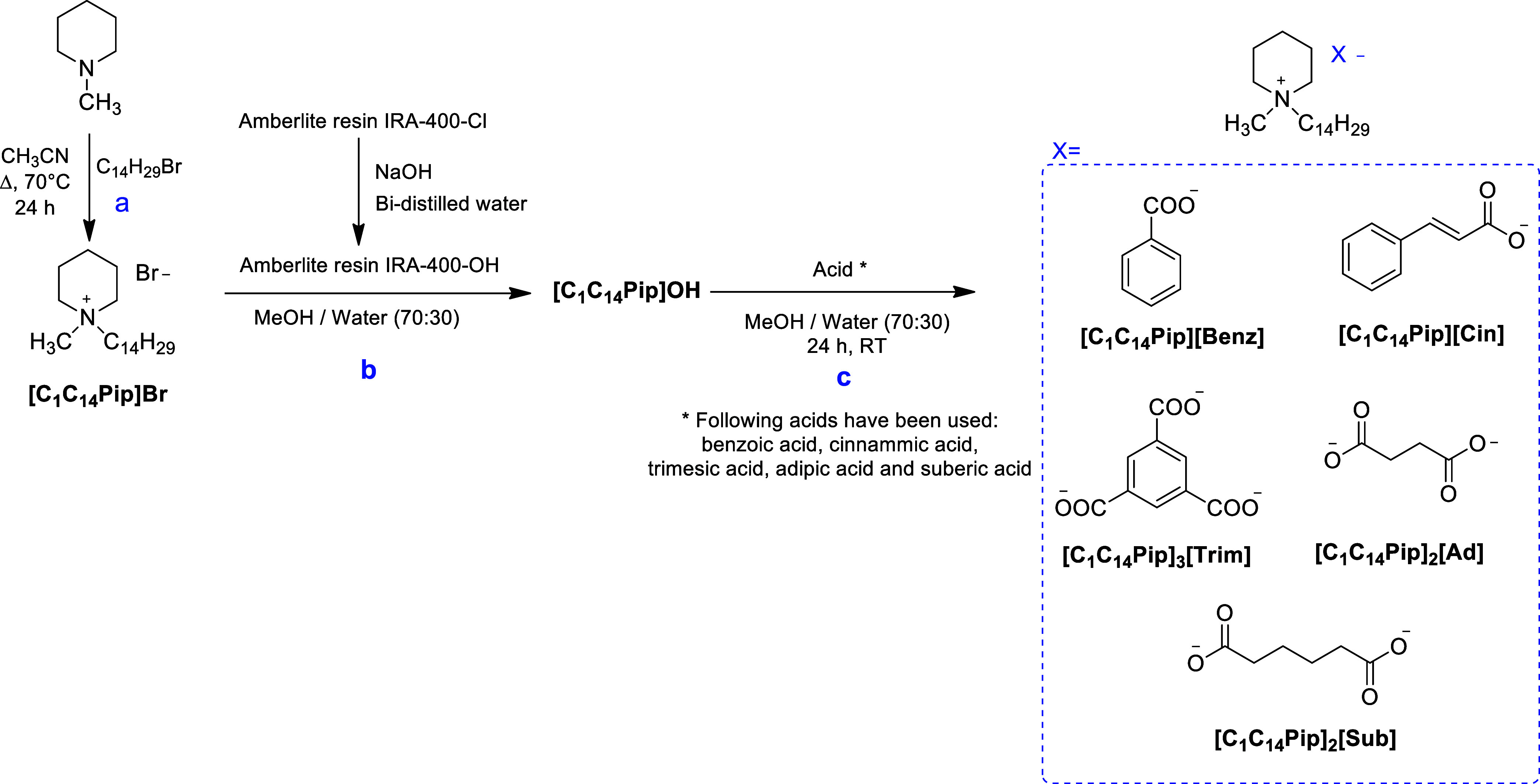
Synthesis of 1-Methyl-1-Tetradecyl-Piperidinium Salt (path a) and its Conversion Via Anion Exchange on Amberlite Resin (path b and c) to Yield Different Piperidinium Salts

New salts **[C**_**8**_ **C**_**2**_ **OHIm]­Br** and **[BzC**_**8**_ **Im]­Br**, based on the imidazolium cation, were prepared from 1-(2-Hydroxyethyl)-imidazole (Scheme 4) or 1-octyl-imidazole (Scheme 5) through reaction with 1-bromooctane and benzyl bromide, respectively.

**Scheme 4:**
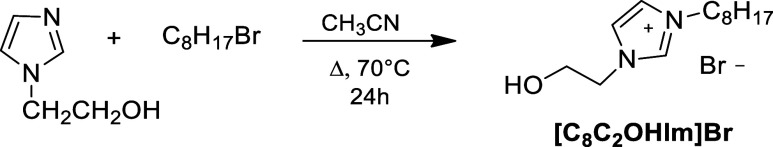
Synthesis of 1-Hydroxyethyl-3-Octyl Imidazolium Bromide Derivative

**Scheme 5:**
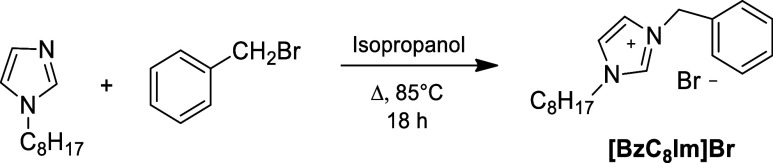
Synthesis of 1-Octyl-3-Benzyl Imidazolium Bromide Derivative

Dicationic salts with aromatic spacer were synthesized as illustrated in Scheme 6. The nature of the cationic head groups, the counterions (bromide, docusate, and tosylate), and the isomeric substitution pattern were varied, preparing *ortho*-, *meta*-, and *para*-isomers. Specifically, 1-octyl-imidazole was reacted with *ortho*-, *meta*-, or *para*-dibromo-xylene in isopropanol to yield the corresponding **[** ***o*** **-xyl­(C**_**8**_ **Im)**_**2**_ **]­Br**_**2**_, **[** ***m*** **-xyl­(C**_**8**_ **Im)**_**2**_ **]­Br**_**2**_, and **[** ***p*** **-xyl­(C**_**8**_ **Im)**_**2**_ **]­Br**_**2**_ isomers (Scheme 6, **path a**). Under the same reaction conditions, **[** ***o*** **-xyl­(C**_**12**_ **Im)**_**2**_ **]­Br**_**2**_ and **[** ***o*** **-xyl­(C**_**1**_ **C**_**1**_ **C**_**8**_ **N)**_**2**_ **]­Br**_**2**_ were obtained from α,α-dibromo-*o*-xylene and either 1-dodecyl-1H-imidazole or *N,N*-dimethyloctylamine, respectively (Scheme 6, path a).

**Scheme 6:**
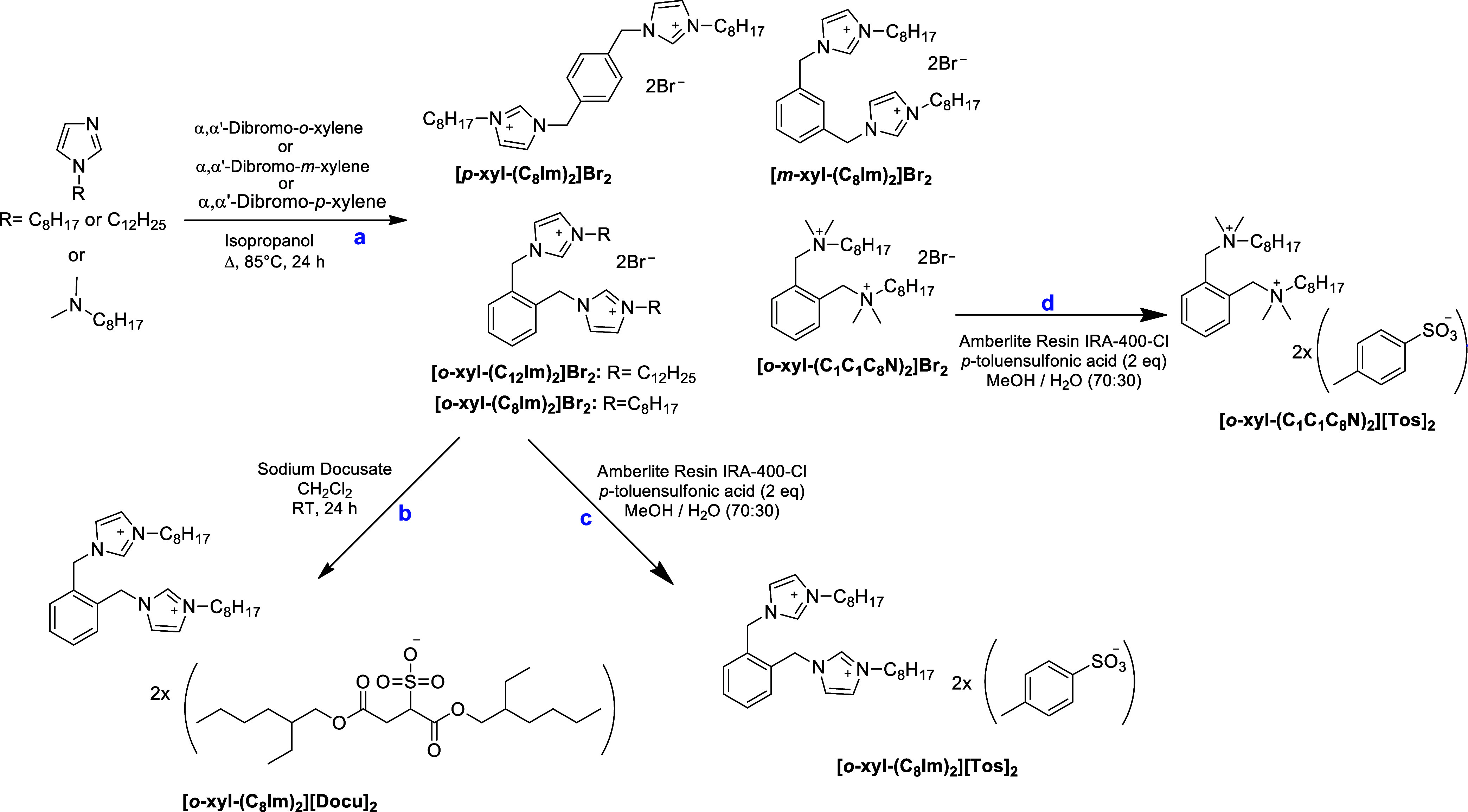
Synthesis of Imidazolium- and Alkylammonium-based Dicationic Derivatives with Bromide Anion (path a) and Anion Exchange Protocol with Sodium Docusate (path b) or *p*-Toluensulfonic acid (Path c and d)

An anion exchange reaction between **[** ***o*** **-xyl­(C**_**8**_ **Im)**_**2**_ **]­Br**_**2**_ and dioctyl sodium sulfosuccinate salt (sodium docusate) in dichloromethane afforded **[** ***o*** **-xyl­(C**_**8**_ **Im)**_**2**_ **]­[Docu]**_**2**_ (Scheme 6, **path b**). Similarly to the procedure described for the piperidinium compounds, anion exchange on Amberlite resin IRA400 (chloride form) allowed the preparation of **[** ***o*** **-xyl­(C**_**8**_ **Im)**_**2**_ **]­[Tos]**_**2**_ and **[** ***o*** **-xyl­(C**_**1**_ **C**_**1**_ **C**_**8**_ **N)**_**2**_ **]­[Tos]**_**2**_ from the corresponding bromide salts and *p*-toluenesulfonic acid (Scheme 6, **paths c** and **d**).

### Thermogravimetric Analysis (TGA)

Thermogravimetric Analysis (TGA) with ramped temperature was used to evaluate the short-term stability of salts. The TGA and derivative thermogravimetric (DTG) curves are shown in Figure S1, with two representative traces provided in Figure 1.

**Figure 1:**
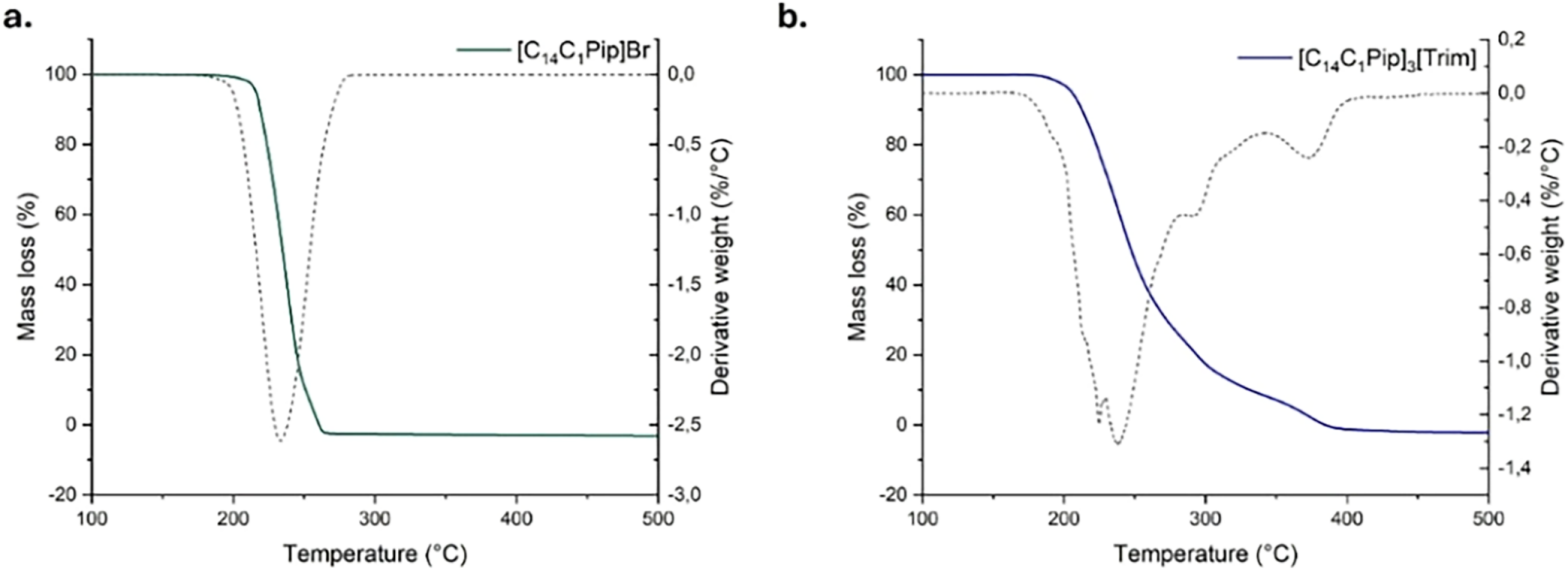
Representative thermogravimetric (TGA) and derivative thermogravimetric curves (DTG) of **[C**_**1**_ **C**_**14**_ **Pip]Br (a)** and **[C**_**1**_ **C**_**14**_ **Pip]­[Trim] (b)**.

Three thermal parameters, commonly used to describe thermal stability, are listed in Table S1. The *T*_onset_ value is obtained from the intersection of the baseline of zero weight loss and the tangent of the weight vs. temperature curve as degradation occurs. Degradation often begins before the *T*_onset_ temperature. Therefore, the temperature corresponding to the 5% weight loss (*T*_d_) and the maximal decomposition temperature obtained from the peak of the derivative TG (DTG) curves (*T*_peak_) are preferred. When multiple peaks appear in the DTG curves, the temperature of the most intense peak is considered for analysis.

All analyzed salts exhibited good thermal stability, with thermal decomposition parameters following the order: *T*_d_ < *T*_onset_ < *T*_peak_. Figure 2 compares the *T*_peak_ values, which range from 221.9 °C for **[C**_**14**_ **C**_**1**_ **Pip]**_**2**_ **[Sub]** to 339.7 °C for **[o-xyl­(C**_**8**_ **Im)**_**2**_ **]­[Tos]**_**2**_.

**Figure 2:**
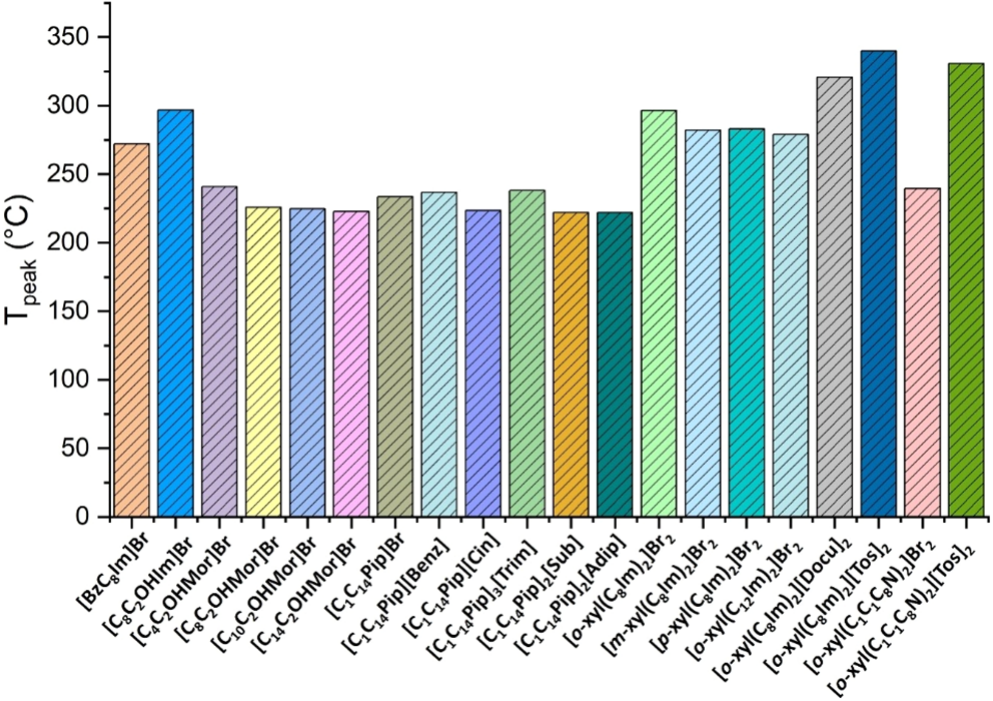
Representative histograms of *T*_peak_ values obtained at the maximum peak of derivative thermogravimetric (DTG) curves of salts.

Morpholinium-based derivatives decomposed in one step, showing the lowest thermal stability among all analyzed compounds.^41^ The **[C**_**4**_ **C**_**2**_ **OHMor]­Br** salt exhibited enhanced thermal stability, indicated by a higher *T*_peak_ value, which decreased when the alkyl chain was extended to eight carbon atoms and then remained nearly constant with further chain elongation (Table S1, entries 3–6).

This result is consistent with previous studies reporting lower stability for imidazolium, pyrrolidinium, pyridinium, and piperidinium salts with longer alkyl chains, likely due to increased conformational flexibility, decreased intramolecular cation–anion electrostatic interactions, and enhanced stability of carbocations and carbon radicals formed during the degradation process.^42,43^

The derivative **[C**_**1**_ **C**_**14**_ **Pip]­Br**, maintaining the same alkyl chain length and anion as **[C**_**14**_ **C**_**2**_ **OHMor]­Br**, showed increased stability (Table S1, cfr entries 6 and 7). Although the influence of the hydroxyethyl chain on the stability of ionic liquids is still unclear, in this case, its presence, together with the morpholinium cation, reduced the degradation temperature.^44^ Therefore, it can be concluded that hydroxyethyl functionalization promotes easier decomposition properties.^45^

When the bromide anion of **[C**_**1**_ **C**_**14**_ **Pip]­Br** is substituted with carboxylate anions, the following stability order was observed: [Trim] ≈ [Benz] > Br > [Cin] > [Adip] ≈ [Sub] (Table S1, entries 7–12). This finding confirms the prominent role of the anion in determining stability.^46^ The high nucleophilicity of the bromide anion, compared to the trimesate and benzoate anions, could induce thermal decomposition of **[C**_**1**_ **C**_**14**_ **Pip]­Br** via nucleophilic cation attack, cleavage of the C–N bond, and dealkylation through an SN_2_ mechanism.^47^ Moreover, as reported in a previous study, thermal stability increases from aliphatic (suberate and adipate) to aromatic (cinnamate, trimesate, and benzoate) carboxylate anions, likely due to the presence of π-stacking interactions.^48^

Except for the bromide and cinnamate anions, the TGA profile indicated a multistep degradation process (Figure S1). The lower degradation temperatures of **[C**_**1**_ **C**_**14**_ **Pip]­[Cin]**, **[C**_**1**_ **C**_**14**_ **Pip]**_**2**_ **[Ad]**, and **[C**_**1**_ **C**_**14**_ **Pip]**_**2**_ **[Sub]** salts, compared to the bromide derivative, are consistent with previous results showing lower thermal stability for ILs bearing carboxylate anions (Table S1, entries 7, 9, 11 and 12).^49^

Furthermore, the incorporation of an aliphatic spacer between the carboxylate moiety and the aromatic ring, along with the presence of a more extended π-conjugated system, leads to a decreased thermodynamic stability of the salt **[C**_**1**_ **C**_**14**_ **Pip]­[Cin]** compared to **[C**_**1**_ **C**_**14**_ **Pip]­[Benz]** (Table S1, entries 8–9). This destabilizing effect of the aliphatic spacer was also evident for the adipate and suberate anions, which exhibited the lowest stability among the piperidinium salts investigated (Table S1, entries 11–12). In contrast, the length of the aliphatic spacer does not appear to have a significant effect, as adipate and suberate anions exhibit comparable *T*_peak_ values.

According to several studies, the presence of the imidazolium ring in **[C**_**8**_ **C**_**2**_ **OHIm]­Br** and **[BzC**_**8**_ **Im]­Br** confers higher stability when compared to morpholinium and piperidinium derivatives discussed earlier (Table S1, entries 1–2).^44,50,51^ The incorporation of an aromatic cationic group in the **[C**_**8**_ **C**_**2**_ **OHIm]­Br** salt leads to a pronounced improvement in thermal stability compared to **[C**_**8**_ **C**_**2**_ **OHMor]­Br**, although both compounds possess identical octyl and hydroxyethyl substituents (Table S1, cfr entries 2 and 3).

Furthermore, the lower stability of **[BzC**_**8**_ **Im]­Br** with an aromatic substituent, compared to **[C**_**8**_ **C**_**2**_ **OHIm]­Br** with a hydroxyethyl chain, aligns with previous findings reporting lower thermal stability for aromatic-containing ionic liquids compared to their aliphatic counterparts.^52,53^

All di-imidazolium derivatives were found to be more stable than the monocationic salts. The enhancement in thermal stability upon introduction of a second cationic headgroup is clearly evident when comparing the *T*_peak_ values of the monocationic **[BzC**_**8**_ **Im]­Br** with the corresponding dicationic isomers (Table S1, entries **1** and **13–15**). Notably, the increase in *T*_peak_ ranges from 10 °C for the *meta*- and *para*-substituted isomers to 20 °C for the *ortho*-substituted derivative.

Among the regio-isomers, the ortho-isomer **[** ***o*** **-xyl­(C**_**8**_ **Im)**_**2**_ **]­Br**_**2**_ exhibited the highest thermal stability. This behavior is likely related to the spatial proximity of the imidazolium rings, which may enable the hydrogen atoms at the C2 position to participate in hydrogen bonding with the bromide anions (Br···C2H). Such interactions are expected to promote a more compact and rigid molecular arrangement, thereby enhancing crystal packing and reducing chain mobility upon heating.

Interestingly, as with morpholinium, extending the alkyl side chain to 12 carbon atoms reduces the stability (Table S1, cfr entries 13 and 16). However, this variation is minimal compared to the marked difference observed when replacing the highly coordinating, hydrophilic, and nucleophilic bromide anion with docusate and tosylate, which share the same sulfate anionic head (Table S1, entries 17–18). The **[** ***o*** **-xyl­(C**_**8**_ **Im)**_**2**_ **]­[Tos]**_**2**_ and **[** ***o*** **-xyl­(C**_**8**_ **Im)**_**2**_ **]­[Docu]**_**2**_ salts exhibited high stability, with *T*_peak_ values of 339.7 and 320.9 °C, respectively. The higher thermal stability conferred by the tosylate anion may be attributed to its smaller volume compared to docusate and higher surface charge density, leading to stronger cation–anion interactions. In contrast, a previous study comparing the electrostatic potential maps of the docusate anion with other organic anions revealed a lower charge density concentrated in the central region of the molecule, resulting in fewer negative sites on its electrostatic surface.^54^ This observation may explain the weaker cation–anion electrostatic interactions and the lower thermal stability observed in salts containing the docusate anion.

The alkylammonium salt **[** ***o*** **-xyl­(C**_**1**_ **C**_**1**_ **C**_**8**_ **N)**_**2**_ **]­Br**_**2**_ displayed the lowest thermal stability among the examined geminal quaternary ammonium salts (Gemini QAS) (Table S1, entry 19). Nonetheless, consistent with previously observed trends, thermal stability was markedly enhanced upon anion exchange from bromide to tosylate, as evidenced by the improved stability of **[** ***o*** **-xyl­(C**_**1**_ **C**_**1**_ **C**_**8**_ **N)**_**2**_ **]­[Tos]**_**2**_ (Table S1, entry 20).

### Differential Scanning Calorimetry Analysis (DSC)

The thermal behavior of synthesized salts and solid–liquid transition temperatures were evaluated by differential scanning calorimetry (DSC) measurements. Table 1 reports thermodynamic parameters obtained from DSC thermograms (Figure S2), whereas a typical DSC trace is shown in Figure 3.

**Figure 3:**
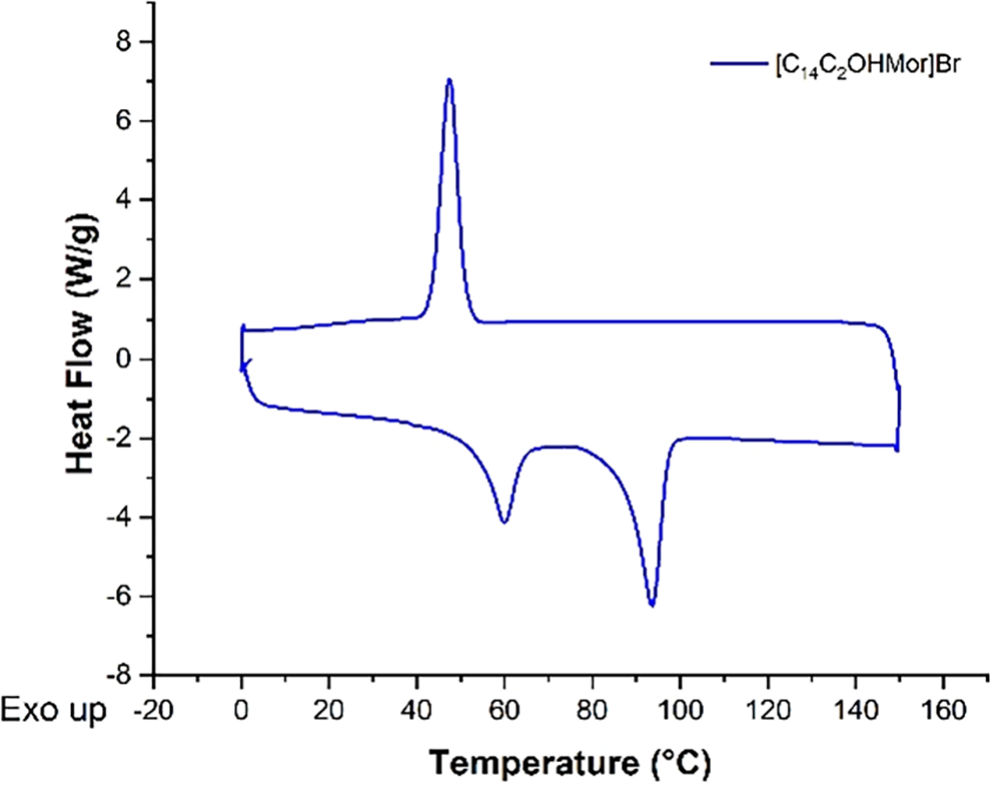
Representative DSC trace of **[C**_**14**_ **C**_**2**_ **OHMor]­Br** salt.

**Table 1:** Thermodynamic Parameters Obtained from DSC Thermograms of Synthesised Salts

entry	sample name	*T*_ss_	Δ*H*_ss_ (kJ/mol)	*T*_m_ (°C)	Δ*H*_m_ (kJ/mol)	*T*_c_ (°C)	Δ*H*_c_ (kJ/mol)
1	[C_4_C_2_OHMor]Br	120.70	11.06	142.42	21.79	106.48	23.68
2	[C_10_C_2_OHMor]Br	8.21	13.00	67.44	14.00	39.45	0.28
3	[C_12_C_2_OHMor]Br	39.56	6.34	87.67	18.76	48.11	5.20
4	[C_14_C_2_OHMor]Br	53.92	11.86	87.23	20.52	50.95	19.81
5	[C_1_C_14_Pip]Br	35.31	0.21	52.17	1.33	64.46	2.03
6	[C_1_C_14_Pip][Benz]	40.93	5.78	117.18	23.15	42.52	8.03
7	[C_1_C_14_Pip]_3_[Trim]			86.60	8.95	63.38	31.38
8	[C_1_C_14_Pip][Cin]	23.41	16.08	102.45	47.48	58.59	0.39
9	[C_1_C_14_Pip]_2_[Sub]			42.38	1.70	45.73	16.32
10	[C_1_C_14_Pip]_2_[Adip]	33.06	1.98	53.06	11.69	44.30	20.98
11	[*o*-xyl(C_12_Im)_2_]Br_2_			50.29	55.71		
12	[*o*-xyl(C_8_Im)_2_]Br_2_			166.66	23.66	106.73	11.98
13	[*m*-xyl(C_8_Im)_2_]Br_2_			142.48	33.18		
14	[*p*-xyl(C_8_Im)_2_]Br_2_	53.49	4.57	100.56	8.08		
15	[*o*-xyl(C_8_Im)_2_][Tos]_2_			115.06	33.13	77.43	22.70
16	[*o*-xyl(C_1_C_1_C_8_N)_2_]Br_2_	24.22	2.78	51.20	4.28	96.37	3.55
17	[*o-*xyl(C_1_C_1_C_8_N)_2_][Tos]_2_			141.26	3.72	110.95	19.68

The solid–solid transition temperature (*T*_ss_) and the melting temperature (*T*_m_) were identified as the points of maximum heat flow associated with endothermic peaks, while the crystallization temperature (*T*_c_) was determined based on the corresponding exothermic peak. Moreover, the enthalpy changes associated with the solid–solid transition (Δ*H*_ss_), melting (Δ*H*_m_), and crystallization (Δ*H*_c_) processes were determined by integrating the corresponding peak areas in the DSC thermograms

Monocationic compounds showed multiple endothermic transitions, detecting the existence of thermotropic polymorphism with a nonreversible solid–solid transition in the first heating cycle, at *T*_ss_ temperature (Figure 3 and Table 1).^55^ As an exception, salts **[C**_**1**_ **C**_**14**_ **Pip]­Br, [C**_**1**_ **C**_**14**_ **Pip]­[Benz], [C**_**12**_ **C**_**2**_ **OHMor]­Br**, and **[C**_**14**_ **C**_**2**_ **OHMor]­Br** showed a solid–solid transition also in the second heating cycle (Figure S2). Moreover, for most salts, as expected, the crystallization temperature *T*_c_ does not exactly match the melting temperature *T*_m_. The difference between the melting temperature and the crystallization temperature in salts is primarily attributed to supercooling, which delays the formation of the crystalline lattice during cooling. This phenomenon is influenced by factors such as the cooling rate and the availability of nucleation sites.^56^

Morpholinium derivatives **[C**_**10**_ **C**_**2**_ **OHMor]­Br**, **[C**_**12**_ **C**_**2**_ **OHMor]­Br**, and **[C**_**14**_ **C**_**2**_ **OHMor]­Br** show an endothermic peak related to the melting process at a temperature below 100 °C, falling in the IL class of compounds. No peaks could be detected for the room temperature ionic liquid **[C**_**8**_ **C**_**2**_ **OHMor]­Br**. In particular, side chains of eight or ten carbons significantly decrease the melting point, while further elongation leads to a new increase in melting temperature for compounds **[C**_**12**_ **C**_**2**_ **OHMor]­Br** and **[C**_**14**_ **C**_**2**_ **OHMor]­Br** (Table 1, entries 1–4). A similar trend was observed for the enthalpy changes associated with the melting process. This finding can be explained by the increase in van der Waals interactions between alkyl long chains that contribute to the crystal ordering.^57^

In a previous study, *N*-alkyl-methylmorpholinium salts with a bromide anion and comparable alkyl chain length exhibited a higher melting temperature than those examined in the present work.^58^ This evidence suggests the prominent role of the hydroxyethyl side-chain in enhancing ionic liquid properties. The same study showed the lowest melting point for *N,N*-dihydroxyethylmorpholinium bromide despite its high structural symmetry, pointing toward a more stable crystal structure and higher melting points.

The salt **[C**_**4**_ **C**_**2**_ **OHMor]­Br** with a shorter alkyl chain showed a high melting point, above 100 °C, with a higher Δ*H*_m_ value. Probably the shorter alkyl chain allows more efficient ion–ion packing and a stable crystal lattice structure with a consequent increase in melting temperature (Table 1, entry 1).^59,60^

Analysis of the TGA and DSC results for morpholinium derivatives allowed determination of the liquid-existence range, defined by the melting temperature as the lower limit and the thermal decomposition temperature as the upper limit (Δ*T* = *T*_d_ – *T*_m_). This temperature range is widest for the **[C**_**10**_ **C**_**2**_ **OHMor]­Br** derivative (Δ*T* = 130 °C) and becomes narrower with the extension of the alkyl chain in **[C**_**14**_ **C**_**2**_ **OHMor]­Br** (Δ*T* = 106 °C). The derivative **[C**_**1**_ **C**_**14**_ **Pip]­Br** with the same alkyl chain and bromide anion shows a lower *T*_m_ than the salt **[C**_**14**_ **C**_**2**_ **OHMor]­Br** (52.17 °C vs. 87.23 °C). Also, the value of enthalpy variation is very low compared to morpholinium cation. In this last, the hydroxyethyl chain and oxygen on the morpholinium cation likely represent acceptor and donor points of hydrogen bonds that raise the melting point.^61^

Among the piperidinium salts, the melting temperatures (*T*_m_) increased in the following order: **[C**_**1**_ **C**_**14**_ **Pip]**_**2**_ **[Sub]** < **[C**_**1**_ **C**_**14**_ **Pip]­Br** < **[C**_**1**_ **C**_**14**_ **Pip]**_**2**_ **[Adip]** < **[C**_**1**_ **C**_**14**_ **Pip]**_**3**_ **[Trim]** < **[C**_**1**_ **C**_**14**_ **Pip]­[Cin]** < **[C**_**1**_ **C**_**14**_ **Pip]­[Benz] (** Table 1, entries 5–10). The combination of *N-*methyl-tetradecyl-piperidinium cation with hydrophilic anions (Br, suberate, and adipate) promotes ionic liquids properties. Conversely, the replacement with aromatic-type anions (benzoate, trimesate, and cinnamate) leads to a higher melting temperature (above 100 °C for [C_1_C_14_Pip]­[Cin] and [C_1_C_14_Pip]­[Benz]). This behavior can be explained by the numerous π-stacking interactions between anions.^62^

In **[C**_**1**_ **C**_**14**_ **Pip]**_**3**_ **[Trim]** derivative, the presence of the piperidinium cation in a favorable stoichiometric ratio may exert a disordering effect on the crystalline structure by disrupting π–π interactions between trimesate anions. As a result, this compound exhibits typical features of ionic liquids, including a lower melting temperature and reduced enthalpy of fusion when compared to other piperidinium-based salts containing aromatic anions, such as **[C**_**1**_ **C**_**14**_ **Pip]­[Cin]** and **[C**_**1**_ **C**_**14**_ **Pip]­[Benz] (** Table 1, cfr entry 7 with 6 and 8).

No endothermic transition in the DSC trace was identified for room temperature ionic-liquid **[** ***o*** **-xyl­(C**_**8**_ **Im)**_**2**_ **]­[Docu]**_**2**_. Other dicationic salts, with large structure and high symmetry, showed melting temperatures above 100 °C, except for **[** ***o*** **-xyl­(C**_**12**_ **Im)**_**2**_ **]­Br**_**2**_.^63^ This last showed an intense endothermic transition around 55 °C only in the first cycle. It is plausible that a long alkyl chain with several rotational degrees of freedom breaks the packing created by van der Waals interactions with other alkyl chains and also interferes with cation–anion Coulomb interactions.^64^

A comparison of the DSC traces of **[** ***o*** **-xyl­(C**_**8**_ **Im)**_**2**_ **]­Br**_**2**_ and **[** ***o*** **-xyl­(C**_**8**_ **Im)**_**2**_ **]­[Tos]**_**2**_ (Table 1, entries 12 and 15) reveals that substitution of bromide with the bulkier, less tightly coordinating tosylate anion leads to a slight decrease in melting temperature. This effect can be attributed to the reduced lattice order and weaker ionic interactions imparted by the tosylate anion, which lowers the thermal energy required for the phase transition.^65^ When the bromide anion of ionic liquid **[** ***o*** **-xyl­(C**_**1**_ **C**_**1**_ **C**_**8**_ **N)**_**2**_ **]­Br**_**2**_ was substituted by tosylate anion, an opposite trend was observed with an increase of melting temperature (Table 1, entries 16–17).

### Antibacterial Evaluation and Structure–Activity Relationship Study

#### Antibacterial Activity of Morpholinium-based Salts

Several works reported the lower toxicity of aliphatic cations (i.e., morpholinium, piperidinium or pyrrolidinium) compared to aromatic ones, such as imidazolium, which is extensively studied for antibacterial applications.^8,28,66,67^ Particularly, among cyclic cations, morpholinium showed low toxicity against cell cultures, bacteria, and algae.^68,69^ Furthermore, the introduction of an oxygenated side chain significantly reduced the toxicity of 3-methyl-imidazolium and *N*-methyl-morpholinium cations compared to 1-butyl-3-methyl imidazolium and *N,N*-butyl-methyl-morpholinium, respectively.^39,68^

The bacterial growth of the *Pseudomonas delhiensis* PS27 strain was not impaired by the treatment with diverse concentrations (ranging from 100 to 400 μg/mL) of the morpholinium derivative **[C**_**4**_ **C**_**2**_ **OHMor]­Br**, which features a hydroxyethyl chain and a short alkyl one (Figure 4a). By increasing the alkyl chain up to eight carbon atoms (i.e., **[C**_**8**_ **C**_**2**_ **OHMor]­Br**), *Pseudomonas* cells showed an impaired growth starting from 200 μg/mL onward (Figure 4a). The enhancement of the antibacterial efficacy was successfully achieved by lengthening the alkyl chain up to 14 carbon atoms, as in the case of **[C**_**14**_ **C**_**2**_ **OHMor]­Br**, which, at a concentration as low as 75 μg/mL (ca. 184 μM), corresponded the minimal inhibitory concentration (MIC) – i.e., the lowest concentration of an antimicrobial agent that inhibits the visible growth of a microorganism after a specified incubation period – of this biocide (Figure 4b).

**Figure 4:**
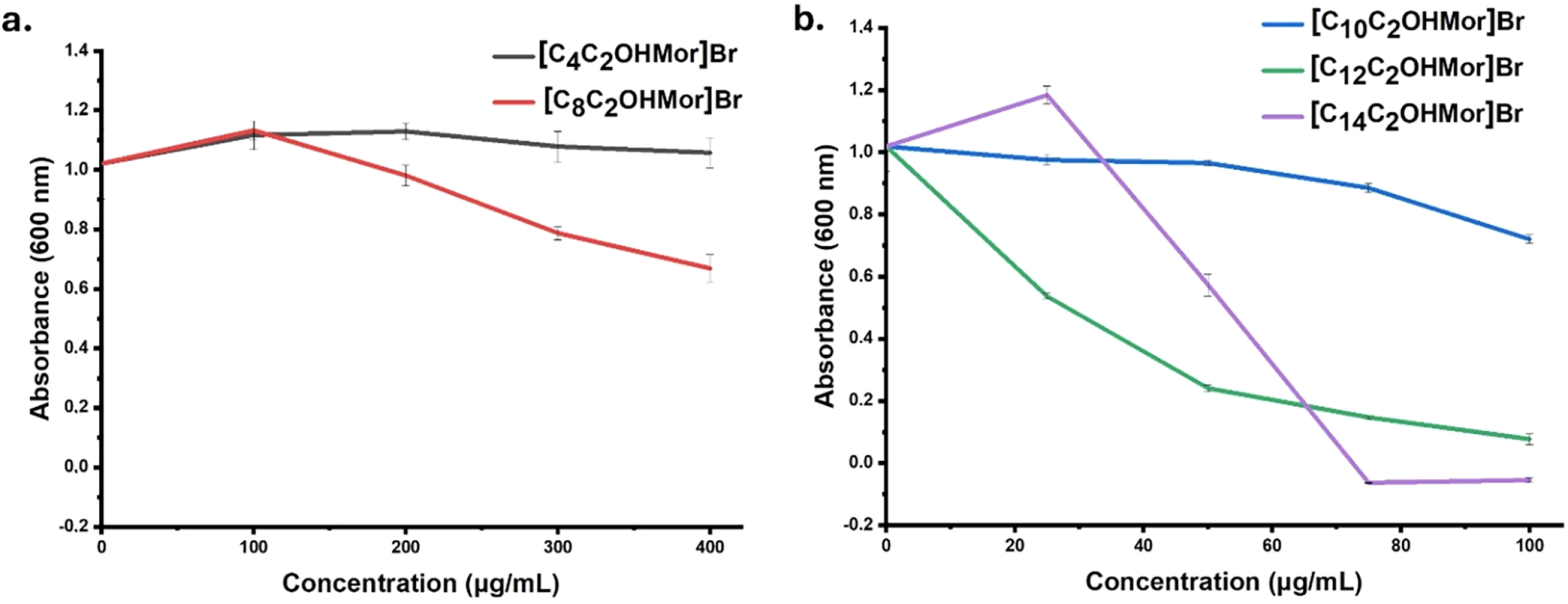
Growth inhibition assay of the *P*. *delhiensis* PS27 strain in the presence of various concentrations of hydroxyl-functionalized morpholinium-based derivatives. (a) Growth response (OD600) to increasing concentrations of **[C**_**4**_ **C**_**2**_ **OHMor]­Br** and **[C**_**8**_ **C**_**2**_ **OHMor]­Br**, while panel (b) reports on the inhibitory effect of **[C**_**10**_ **C**_**2**_ **OHMor]­Br**, **[C**_**12**_ **C**_**2**_ **OHMor]­Br**, and **[C**_**14**_ **C**_**2**_ **OHMor]­Br**.

This result is of some importance considering the environmental resilience of the tested *Pseudomonas* strain, adding to the few studies relating to the antimicrobial activity of salts based on *N*-hydroxyethyl-morpholinium cation substituted with long alkyl side chains.

Rahimov et al. previously investigated the effect of hydroxyethyl substitution on the antibacterial activity of alkylammonium salts,^70^ while in a separate study, they examined the cation 1-(4-morpholinyl)-2-propanol bearing a tetradecylalkyl side chain. This latter compound, which differs from **[C**_**14**_ **C**_**2**_ **OHMor]­Br** in the position of the OH group within the side chain, had shown good antibacterial activity against the Gram-positive strain *Staphylococcus aureus* and the Gram-negative *Escherichia coli* and *P. aeruginosa* strains.^71^ Also, Soukup et al. investigated the antibacterial activity of **[C**_**14**_ **C**_**2**_ **OHMor]­Br** and its analogue with a longer alkyl chain against a panel of six Gram-negative bacterial strains.^72^

#### Antibacterial Properties of Piperidinium-Based Salts

The *N,N*-methyl-tetradecyl-piperidinium derivative **[C**_**1**_ **C**_**14**_ **Pip]­Br** was more toxic than **[C**_**14**_ **C**_**2**_ **OHMor]­Br** salt, although these biocides feature an equal alkyl chain length (Figure 5). Indeed, to achieve the same inhibitory effect (i.e., MIC), it was sufficient to add three times less **[C**_**1**_ **C**_**14**_ **Pip]­Br** (i.e., 25 μg/mL, corresponding to ca. 66 μM) compared to **[C**_**14**_ **C**_**2**_ **OHMor]­Br**. This evidence suggests that the morpholinium cation and the *N*-hydroxyethyl chain represent structural motifs that minimize the toxic effects of morpholinium-based derivatives on bacterial cells. Therefore, the well-known dependence of the cytotoxicity on cation’s lipophilicity can also be confirmed for *P. delhiensis* PS27 strain.^27,73,74^

**Figure 5:**
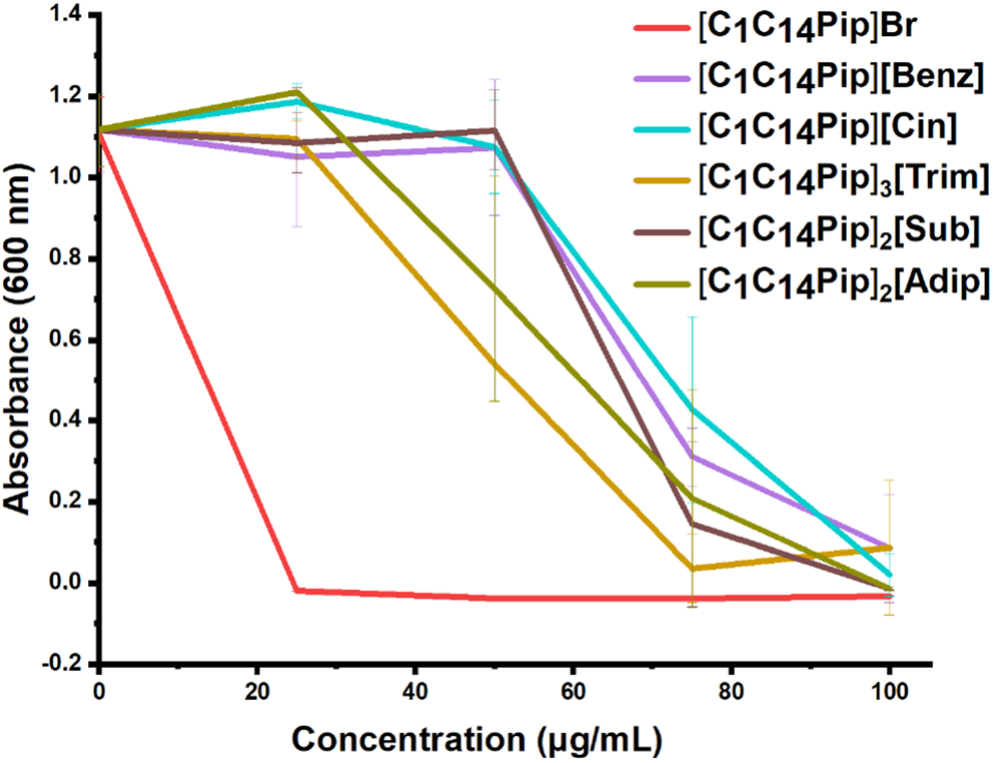
Growth inhibition assay of the *P*. *delhiensis* PS27 strain in the presence of various concentrations of *N,N*-methyl-tetradecyl piperidinium-based derivatives.

Despite differences in the MIC values of **[C**_**14**_ **C**_**2**_ **OHMor]­Br** and **[C**_**1**_ **C**_**14**_ **Pip]­Br**, these biocides represent good candidates for antibacterial applications, considering that their inhibitory effect on bacterial proliferation is in line with or even better than other compounds previously tested.^75^

In the search for antibacterial agents with minimal toxicity toward nontarget biological systems, such as mammalian cell lines, the **[C**_**14**_ **C**_**2**_ **OHMor]­Br** derivative stands out as a promising candidate for further investigation. Its molecular architecture, characterized by an oxygenated side chain and a morpholinium ring, includes structural elements known in the literature to enhance compatibility with mammalian cells while preserving antibacterial effectiveness.^76,77^

The role played by the anion in the antimicrobial activity was analyzed by synthesizing a set of new *N*-methyl-tetradecyl-piperidinium-based salts differing from **[C**_**1**_ **C**_**14**_ **Pip]­Br** for the presence of organic carboxylate anion (Scheme 3). To the best of our knowledge, this is the first time that the antibacterial activity of the *N,N*-methyl-tetradecyl-piperidinium cation has been evaluated in combination with these anions. Furthermore, no previous studies have reported on the antibacterial activity of these anions. All tested salts in Scheme 3 are less active than the bromide precursor **[C**_**1**_ **C**_**14**_ **Pip]­Br** (Figure 5). For this last derivative, it is plausible that there is a weaker electrostatic attraction between the anion and the cation. This may be due to the easier solvation of the bromide anion, which is favored by its smaller size and hydrophilicity. Therefore, the piperidinium cation is free to intercalate the cell membrane, causing damage to the cell.^78^

Salts like **[C**_**1**_ **C**_**14**_ **Pip]­[Cin]** and **[C**_**1**_ **C**_**14**_ **Pip]­[Benz]**, having the same cation/anion ratio as **[C**_**1**_ **C**_**14**_ **Pip]­Br**, are toxic only at higher concentrations (Figure 5). On the other hand, the salt **[C**_**1**_ **C**_**14**_ **Pip]**_**3**_ **[Trim]** was found to be the most harmful, followed by **[C**_**1**_ **C**_**14**_ **Pip]**_**2**_ **[Adip]** and **[C**_**1**_ **C**_**14**_ **Pip]**_**2**_ **[Sub]** (Figure 5). All these derivatives show a stoichiometric ratio favoring the cation, confirming the prominent role of cation and side-chain length in antimicrobial activity.^9^

### Antibacterial Activity of Imidazolium-Based Salts

As for imidazolium-based salts, the literature reports on several examples of the biocidal action of the imidazolium cation with particular attention to 1-alkyl-3-methyl-imidazolium. Unlike ammonium cations, imidazolium showed interesting antibacterial and antifungal activity, depending on the length of the alkyl chain.^26,79,80^

The antimicrobial activity evaluation (Figure 6) of the new salts based on the imidazolium cation (Scheme 4–5) showed that the sensitivity of bacterial cells to the cation varies significantly when a hydroxyethyl chain or benzyl substituent is present on the ring. Specifically, **[C**_**8**_ **C**_**2**_ **OHIm]­Br** showed no antibacterial activity up to 100 μg/mL (corresponding to 328 μM), likely due to the hydroxyethyl side chain capable of attenuating the octyl-mediated toxicity, as mentioned earlier for the morpholinium cation. Conversely, the benzyl substitution in **[BzC**_**8**_ **Im]­Br** significantly increases the antimicrobial performance, as highlighted by the progressive decline in bacterial growth, culminating in near-complete inhibition at a biocide concentration of 100 μg/mL (corresponding to 285 μM). Thus, as previously reported by Crnčević et al. testing **[BzC**_**10–14**_ **Im]­Br** salts on other bacterial strains, the alkyl chain elongation on the imidazole cations would be expected to enhance the antibacterial performance.^81^

**Figure 6:**
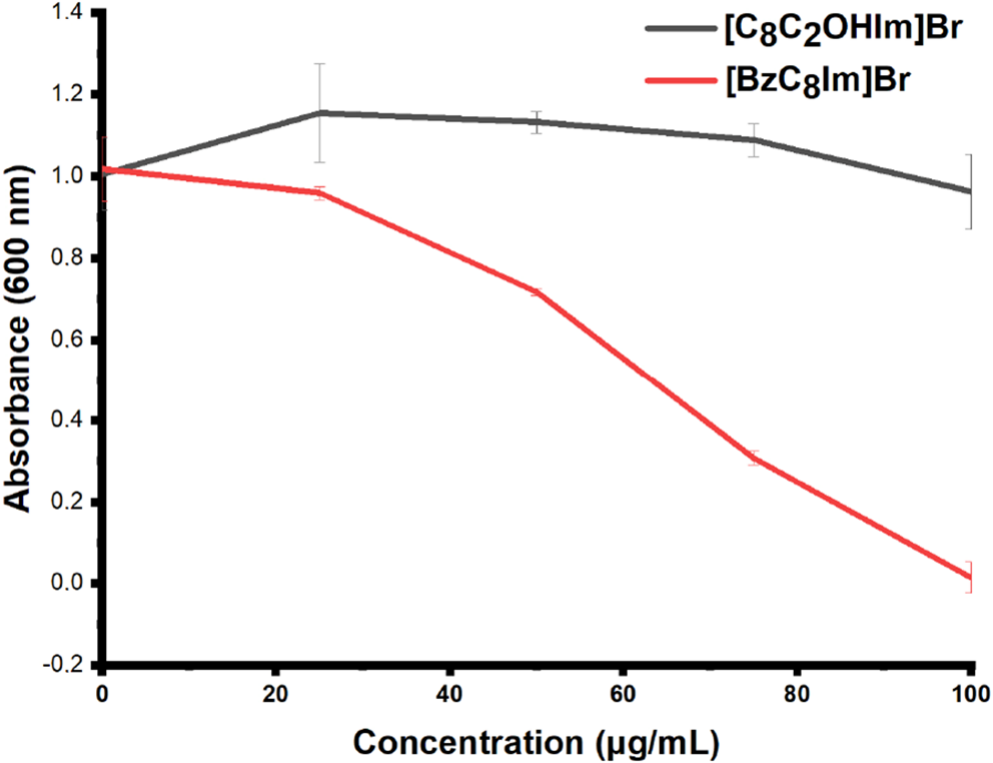
Growth inhibition assay of the *P*. *delhiensis* PS27 strain in the presence of various concentrations of imidazolium-based derivatives.

#### Antibacterial Activity of Dicationic Salts

Building on the structural motif of 3-benzyl-1-octylimidazolium, which proved to be active, the synthesis of dicationic derivatives with an aromatic spacer was envisaged to obtain a more potent antimicrobial compound. (Scheme 6).

In line with expectations, the second cationic head significantly enhanced the antibacterial activity against the tested strain (Figure 7). The isomers **[** ***o*** **-xyl­(C**_**8**_ **Im)**_**2**_ **]­Br**_**2**_, **[** ***m*** **-xyl­(C**_**8**_ **Im)**_**2**_ **]­Br**_**2**_, and **[** ***p*** **-xyl­(C**_**8**_ **Im)**_**2**_ **]­Br**_**2**_ showcased a strong bactericidal action, almost completely inhibiting *Pseudomonas* cell growth at a concentration as low as 25 μg/mL (corresponding to 40 μM). The different isomeric substitutions did not show any substantial differences. On the other hand, the growth inhibition profile of bacterial cells treated with **[** ***o*** **-xyl­(C**_**12**_ **Im)**_**2**_ **]­Br**_**2**_ showed that alkyl chain elongation reduces the antibacterial performance. This may be attributed to the increased conformational freedom, which could hinder interactions with cell membranes.^82^ In this regard, **[** ***o*** **-xyl­(C**_**12**_ **Im)**_**2**_ **]­Br**_**2**_ showed good antimicrobial performances at a concentration of 25 μg/mL (corresponding to 34 μM); instead, higher concentrations of the same biocide resulted in a mitigated toxic effect on bacterial cells (Figure 7). This behavior suggested that, at concentrations above 25 μg/mL, the longer alkyl chain of derivative **[** ***o*** **-xyl­(C**_**12**_ **Im)**_**2**_ **]­Br**_**2**_ may facilitate self-assembly processes such as aggregation and micellization, leading to decreased bioavailability and, consequently, attenuated toxicity.

**Figure 7:**
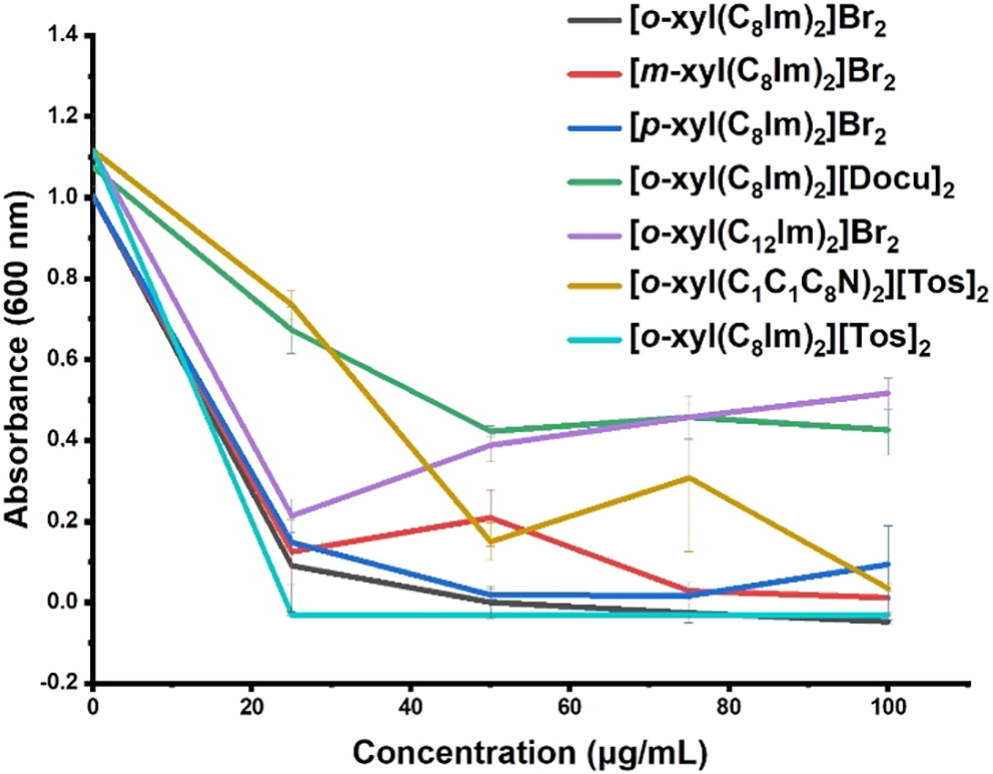
Growth inhibition assay of the *P*. *delhiensis* PS27 strain in the presence of various concentrations of imidazolium-based dicationic derivatives.

To further evaluate the performance of **[** ***o*** **-xyl­(C**_**8**_ **Im)**_**2**_ **]** ^**2+**^ cation, the bromide anion was substituted with the hydrophobic anion docusate (Scheme 6 - path b). Due to its low toxicity, docusate is a widely used additive in the pharmaceutical field. Furthermore, Wylie et al. recently reported its use as an anion in designing phosphonic ILs used as lubricants for slippery liquid-infused porous surfaces (SLIP). These surfaces prevent bacterial colonisation through a dual antiadhesive/antimicrobial approach.^83^

The newly synthesized derivative **[** ***o*** **-xyl­(C**_**8**_ **Im)**_**2**_ **] [Docu]**_**2**_ showed some inhibition of bacterial growth up to 50 μg/mL (corresponding to 38 μM), with absorbance values halved compared to untreated cells (Figure 7). Increasing the concentration did not seem to enhance the antibacterial activity. Thus, the antibacterial activity combined with the ionic liquid properties of **[** ***o*** **-xyl­(C**_**8**_ **Im)**_**2**_ **]­[Docu]**_**2**_ may suggest its use as a lubricant for SLIP surfaces to replace volatile and unstable fluorinated lubricants.

The substitution of the bromide by the tosylate anion did not significantly alter the activity (Figure 7). **[** ***o*** **-xyl­(C**_**8**_ **Im)**_**2**_ **]­[Tos]**_**2**_ and **[** ***o*** **-xyl­(C**_**8**_ **Im)**_**2**_ **]­Br**_**2**_ salts are the most potent dicationic compounds with no additional anion-mediated toxicity.

This result agrees with a previous study in which the calculation of a parameter termed “anion effect ratio” showed that the tosylate anion did not significantly alter the toxicity of imidazolium cations if compared to chloride, chosen as a reference.^84^

Interestingly, replacing the imidazolium with alkylammonium heads in **[** ***o*** **-xyl­(C**_**8**_ **C**_**1**_ **C**_**1**_ **N)**_**2**_ **]­[Tos]**_**2**_ resulted in a compound that was active at higher concentrations (Figure 7). These results confirm the prominent role of the cation rather than the anion concerning the antimicrobial activity of the tested compound. Moreover, according to literature data, the imidazolium cation showed greater antibacterial properties, probably due to the planar nature of the ring.^85^

## Conclusions

This study investigated the synthesis and characterization of a series of quaternary ammonium salts (QASs), including compounds with ionic liquid (IL) properties. These were based on morpholinium, piperidinium, imidazolium, dialkylammonium, and di-imidazolium cations. Structural variations, including alkyl chain length, functionalized side chains, counterion types, and the presence of a second cationic head, significantly influence thermal stability and phase transition behavior.

Specifically, *N*-hydroxyethylmorpholinium-based derivatives with short alkyl chains, *N*-methylpiperidinium compounds bearing aromatic anions, and di-imidazolium salts with tosylate or docusate anions showed greater thermal stability. Furthermore, the introduction of a hydroxyethyl group in morpholinium salts promotes ionic liquid behavior. However, compared to piperidinium analogues, these compounds showed reduced thermal stability and higher melting points, likely due to increased hydrogen bonding and altered crystal packing.

For the first time, the antibacterial activity of these salts was evaluated against the multidrug-resistant Gram-negative strain *Pseudomonas delhiensis PS27*. The molecular design and strategically incorporated structural features aimed at enhancing antimicrobial efficacy while reducing potential toxicity to nontarget organisms, supporting their potential for environmentally safer antibiofilm applications.

A clear structure–activity relationship (SAR) emerged: increasing the alkyl chain length led to a marked improvement in antibacterial efficacy, especially for morpholinium and imidazolium derivatives.

Among the most active compounds, piperidinium-based ionic liquids – **[C**_**1**_ **C**_**14**_ **Pip]­Br** and **[C**_**1**_ **C**_**14**_ **Pip]**_**3**_ **[Trim]** – were identified as highly effective.

The liquid ionic **[C**_**14**_ **C**_**2**_ **OHMor]­Br** also exhibited promising antimicrobial performance. This suggests that its structural features – the morpholinium cation and the *N*-hydroxyethyl side chain – might offer a favorable balance between antimicrobial potency and potential biocompatibility.

Dicationic imidazolium salts, especially **[o-xyl­(C**_**8**_ **Im)**_**2**_ **]­Br**_**2**_, showed strong antibacterial effects at low concentrations, outperforming their monocationic and alkylammonium counterparts. Furthermore, the bacterial growth inhibition assay of tested compounds led to the identification of a new dicationic IL, **[o-xyl­(C**_**8**_ **Im)**_**2**_ **]­[Docu]**_**2**_, which combines ionic liquid properties with potent antimicrobial activity. These findings highlight its potential as a promising candidate for the development of advanced antifouling materials, including slippery liquid-infused porous (SLIP) surfaces.

Overall, this work provides clear guidelines for tuning the physicochemical and biological properties of ILs. The structure–activity relationships identified here offer a valuable foundation for the rational design of new QAS- and IL-based systems for biomedical, antimicrobial, and advanced material applications.

## Supplementary Material



## Notes

Detailed synthetic protocols, ^1^HNMR and ^13^CNMR spectra, differential scanning calorimetry (DSC) and derivative thermogravimetric (TGA-DTG) curves for synthesized QASs; thermal parameters obtained from TGA and DTG profiles (PDF)
