# Supplementary material for: Structurally Tailored Antibacterial Quaternary Ammonium Salts with Ionic Liquid Properties for Antimicrobial Purposes: Design and Thermophysical Insights
Source: ACS Sustain Chem Eng. 2025 Oct 27;13(44):19108–21. doi: 10.1021/acssuschemeng.5c07350 (PMC12610403; doi:10.1021/acssuschemeng.5c07350)
Supplement: Supplementary file 1 [file sc5c07350_si_001.pdf]

Supplementary Information

Structurally Tailored Antibacterial Quaternary Ammonium Salts with Ionic Liquid Properties for Antimicrobial Purposes: Design and Thermophysical Insights.

Paola Marzullo,[†, ‡] Salvatore Marullo,[†] Alessandro Presentato,[†] Enrico Tornatore,[†] Carla Rizzo,[†] Rosa Alduina,[†] Michelangelo Gruttadauria[†, ‡], Francesca D'Anna[†, ‡, *]

[†] *Department STEBICEF, University of Palermo, Viale delle Scienze, Ed. 17, Palermo, Italy*
[‡] *Sustainable Mobility Center (Centro Nazionale per la Mobilità Sostenibile—CNMS), Via Durando 39, Milano, Italy*

*corresponding author: francesca.danna@unipa.it

Number of Pages: 20

Number of Figures: 2

Number of Tables: 1

Content **Page**

- Procedure for the synthesis of morpholinium-based salt [C_4C_2OHMor]Br…………………… S3
- General procedure for the synthesis of morpholinium-based salts [C_8C_2OHMor]Br, [$C_{10}C_2$OHMor]Br, [$C_{12}C_2$OHMor]Br and [$C_{14}C_2$OHMor]Br…………………………………… S3
- Procedure for the synthesis of piperidinium-based salt [C_1C_{14}Pip]Br…………………………… S5
- General procedure for the synthesis of piperidinium-based salts [C_1C_{14}Pip][Benz], [C_1C_{14}Pip]$_3$[Trim], [C_1C_{14}Pip][Cin], [C_1C_{14}Pip]$_2$[Sub] and [C_1C_{14}Pip]$_2$[Adip]………………….. S5
- Procedure for the synthesis of [C_8C_2OHIm]Br salt………………………………………………. S7
- Procedure for the synthesis of [BzC_8Im]Br salt………………………………………………….. S7
- Procedure for the synthesis of dicationic salts [*o*-xyl(C_8Im)$_2$]Br$_2$ and [*o*-xyl($C_8C_1C_1$)$_2$]Br$_2$……… S8
- Procedure for the synthesis of dicationic salts [*o*-xyl(C_{12}Im)$_2$]Br$_2$ ………………………………. S8
- Procedure for the synthesis of dicationic salt [*o*-xyl(C_8Im)$_2$][Docu]$_2$ …………………………… S9
- Procedure for the synthesis of dicationic salts [*o*-xyl(C_8Im)$_2$][Tos]$_2$ and [*o*-xyl($C_8C_1C_1$N)$_2$] [Tos]$_2$ S10
- **Figure S1.** Thermogravimetric (TGA) and derivative thermogravimetric curves (DTG) of synthetised salts……………………………………………………………………….................. S12
- **Table S1.** Thermal parameters of synthetized salts obtained from TGA and DTG profiles……… S15
- **Figure S2**. DSC thermograms of synthetized salts………………………………….................... S16

Procedure for the synthesis of morpholinium-based salt [C_4C_2OHMor]Br

In a two-neck round bottomed flask, 4-butylmorpholine (500 mg, 3.4 mmol) was dissolved in 5 mL of acetonitrile (CH_3CN). In a separate vessel, a stoichiometric amount of 2-bromoethanol (3.4 mmol, 437 mg) was dissolved in 2.5 mL of CH_3CN. The bromoethanol solution was then added dropwise to the 4-butylmorpholine solution under continuous magnetic stirring at room temperature. The resulting mixture was heated to reflux at 70 °C and maintained under these conditions for 24 hours. The solvent was evaporated under reduced pressure, and the crude product was washed three times with acetone (3 × 5 mL) to afford the product.

[C_4C_2OHMor]Br: white solid, yield 13%; ^{1}H-NMR (DMSO) δ ppm: 4.82 (s, 1H, OH), 4.04-4.00 (m, 6H, 2x -$^+$NCH$_2$**CH$_2$**O- and -$^+$NCH$_2$**CH$_2$**OH), 3.71-3.52 (m, 8H, -$^+$N**CH$_2$**CH$_2$CH$_2$CH$_3$, -$^+$N**CH$_2$**CH$_2$OH and 2 x -$^+$N**CH$_2$**CH$_2$O-), 1.81-1.73 (m, 2H, $^+$NCH$_2$**CH$_2$**CH$_2$CH$_3$), 1.48-1.42 (m, 2H, $^+$NCH$_2$ CH$_2$**CH$_2$**CH$_3$), 1.04 (t, 3H, **CH$_3$**); ^{13}C-NMR (DMSO) δ ppm: 60.5, 60.1, 59.3, 59.0, 55.0, 23.1, 19.3, 12.6.

General procedure for the synthesis of morpholinium-based salts [C_8C_2OHMor]Br, [$C_{10}C_2$OHMor]Br, [$C_{12}C_2$OHMor]Br and [$C_{14}C_2$OHMor]Br

In a two-neck round bottomed flask, 4-(2-hydroxyethyl)morpholine (1g, 7.6 mmol) was dissolved in acetonitrile (10 mL/g of 4-(2-hydroxyethyl)morpholine). In a separate flask, a stoichiometric amount of the appropriate bromo alkane (7.6 mmol) was dissolved in 5 mL of CH_3CN and subsequently added dropwise to the 4-(2-hydroxyethyl)morpholine solution, while maintaining the reaction mixture at room temperature under continuous magnetic stirring. The reaction mixture was then refluxed at 70 °C for 24 hours. The solvent was evaporated, and the crude solid product was washed three times with diethyl ether (3 × 10 mL) or cyclohexane (2 x 5mL) and ethyl acetate (2 x 5mL) for **[$C_{12}C_2$OHMor]Br** salt.

[C_8C_2OHMor]Br: orange oil; yield 22%; ^{1}HNMR ($CDCl_3$) δ ppm: 4.19-4.06 (m, 4H, -$^+NCH_2\mathbf{CH_2}O$-), 3.99-3.95 (m, 2H, -$^+NCH_2\mathbf{CH_2}OH$), 3.90-3.88 (m, 4H, $^+N\mathbf{CH_2}CH_2O$-), 3.68-3.64 (m, 2H, $^+N\mathbf{CH_2}CH_2OH$), 3.60-3.55 (m, 2H, $^+\mathbf{N}\mathbf{CH_2}CH_2(CH_2)_5CH_3$), 1.75-1.69 (m, 2H, $^+NCH_2\mathbf{CH_2}(CH_2)_5CH_3$), 1.34-1.20 (m, 10H, $^+NCH_2CH_2\mathbf{(CH_2)_5}CH_3$), 0.84 (t, 3H, $\mathbf{CH_3}$); ^{13}CNMR ($CDCl_3$) δ ppm: 61.0, 60.6, 59.9, 59.2, 55.4, 31.7, 29.1, 26.4, 22.6, 21.9, 14.1.

[$C_{10}C_2$OHMor]Br: yellow oil, yield 30%; ^{1}HNMR ($CDCl_3$) δ ppm: 5.06 (s, 1H, OH), 4.20-4.15 (m, 2H, -$^+NCH_2\mathbf{CH_2}O$-), 3.99 (m, 2H, -$^+NCH_2\mathbf{CH_2}O$-), 3.99-3.91 (m, 6H, -$^+NCH_2\mathbf{CH_2}OH$ and 2 x -$^+N\mathbf{CH_2}CH_2O$-), 3.69-3.65 (m, 2H, $^+N\mathbf{CH_2}CH_2OH$), 3.60-3.55 (m, 2H, $^+N\mathbf{CH_2}CH_2(CH_2)_7CH_3$), 1.76-1.70 (m, 2H, -$^+NCH_2\mathbf{CH_2}(CH_2)_7CH_3$), 1.34-1.23 (m, 14H, -$^+NCH_2CH_2\mathbf{(CH_2)_7}CH_3$), 0.85 (t, 3H, CH_3); ^{13}CNMR ($CDCl_3$) δ ppm: 61.4, 60.7, 59.8, 59.3, 55.4, 31.8, 29.4, 29.2, 29.1, 26.4, 22.6, 21.9, 14.1.

[$C_{12}C_2$OHMor]Br: light pink solid, yield 10%; ^{1}HNMR ($CDCl_3$) δ ppm: 5.12 (t, 1H, OH), 4.24-4.18 (m, 2H, -$^+NCH_2\mathbf{CH_2}O$-), 4.12 (m, 2H, -$^+NCH_2\mathbf{CH_2}O$-), 4.02-3.93 (m, 6H, $^+NCH_2\mathbf{CH_2}OH$ and 2 x___-$^+N\mathbf{CH_2}CH_2O$-), 3.71-3.67 (m, 2H, $^+N\mathbf{CH_2}$ CH_2OH), 3.62-3.55 (m, 2H,$^+\mathbf{N}\mathbf{CH_2}CH_2(CH_2)_9CH_3$),1.80-1.72 (m, 2H, -$^+NCH_2\mathbf{CH_2}(CH_2)_9CH_3$), 1.40-1.25 (m, 18H, -$^+NCH_2CH_2\mathbf{(CH_2)_9}CH_3$) ,0.87 (t, 3H, CH_3);

[$C_{14}C_2$OHMor]Br: white solid, yield 28.8%; ^{1}HNMR (DMSO) δ ppm: 5.29 (s, 1H, OH), 3.93-3.90 (m, 4H, -$^+NCH_2\mathbf{CH_2}O$-), 3.82-3.79 (m, 2H, -$^+NCH_2\mathbf{CH_2}OH$), 3.59-3.56 (m, 2H, -$^+N\mathbf{CH_2}CH_2O$-), 3.53-3.43 (m, 6H, $^+N\mathbf{CH_2}CH_2(CH_2)_{11}CH_3$ and 2 x -$^+N\mathbf{CH_2}CH_2O$-), 1.67-1.60 (m, 2H, -$^+NCH_2\mathbf{CH_2}(CH_2)_{11}CH_3$), 1.29-1.23 (m, 22H, -$^+NCH_2CH_2\mathbf{(CH_2)_{11}}CH_3$), 0.83 (t, 3H, CH_3); ^{13}CNMR ($CDCl_3$) δ ppm: 61.4, 60.7, 59.8, 59.3, 55.5, 31.9, 29.7, 29.5, 29.5, 29.4, 29.2, 26.4, 22.7, 21.9, 14.1.

Procedure for the synthesis of piperidinium-based salt [C_1C_{14}Pip]Br

In a two-neck round bottomed flask, 1-methylpiperidine (500 mg, 5.04 mmol) was dissolved in acetonitrile (5 mL). In a separate flask, a stoichiometric amount of 1-bromotetradecane (5.04 mmol, 1.39 g) was dissolved in 2.5 mL of CH_3CN. The alkyl halide solution was added dropwise to the amine solution under continuous magnetic stirring at room temperature. The resulting mixture was then heated to reflux at 70 °C and stirred for 60 hours. After this time, the solvent was evaporated under reduced pressure, and the crude solid was washed three times with ethyl acetate (3 × 10 mL). The final solid product was then isolated by centrifugation.

[C_1C_{14}Pip]Br: white solid, yield 87%; ^{1}HNMR ($CDCl_3$) δ ppm: 3.81-3.75 (m, 2H, $^+N\mathbf{CH_2}CH_2(CH_2)_{11}CH_3$), 3.67-3.59 (m, 4H, 2 x $^+N\mathbf{CH_2}$), 3.33 (s, 3H, $^+N\mathbf{CH_3}$), 1.94-1.65 (m, 8H, 3 x **- CH_2** Pip and $^+NCH_2\mathbf{CH_2}(CH_2)_{11}CH_3$), 1.38-1.22 (m, 22H, $^+NCH_2CH_2\mathbf{(CH_2)_{11}}CH_3$), 0.84 (t, 3H, CH_3);^{13}CNMR ($CDCl_3$) δ ppm: 62.9, 60.8, 48.4, 31.9, 29.6, 29.5, 29.4, 29.4, 29.3, 29.2, 26.4, 22.6, 22.0, 20.7, 20.2, 14.0.

General procedure for the synthesis of piperidinium-based salts [C_1C_{14}Pip][Benz], [C_1C_{14}Pip]$_3$[Trim], [C_1C_{14}Pip][Cin], [C_1C_{14}Pip]$_2$[Sub] and [C_1C_{14}Pip]$_2$[Adip]

Anion exchange was carried out following a previously reported protocol, using Amberlite IRA-400 resin in the chloride form to obtain salts of [C_1C_{14}Pip]$^+$ bearing different counterions.[83]

For the exchange process, Amberlite IRA-400 resin (1.64 g per mmol of [C_1C_{14}Pip]Br) was packed into a column using distilled water. The resin was converted from the chloride to the hydroxide form by washing with an aqueous solution of sodium hydroxide (10% w/v), prepared by dissolving 52 mg of NaOH per gram of resin. Washing was continued until the eluate reached a neutral pH. Subsequently, the bromide salt [C_1C_{14}Pip]Br was dissolved in a methanol/water binary mixture (70:30, v/v) and passed through the column, using the same solvent mixture as the eluent. The eluate was collected in a flask containing a stoichiometric amount of the target acid, and elution was continued until a neutral pH was observed at the column outlet. The collected solution was stirred at

room temperature overnight. After solvent removal under reduced pressure, the residue was washed with diethyl ether (3 × 10 mL). The resulting solid product was dried under vacuum.

[C_1C_{14}Pip][Benz]: white solid; yield 87%; ^{1}HNMR ($CDCl_3$) δ ppm: 8.05-8.03 (m, 2H, Ar), 7.35-7.29 (m, 3H, Ar), 3.57-3.53 (m, 2H, $^+$N**CH_2**$CH_2(CH_2)_{11}CH_3$), 3.43-3.31 (m, 4H, 2 x $^+$N**CH_2** Pip), 3.18 (s, 3H, $^+$N**CH_3**), 1.80-1.56 (m, 8H, 3 x **- CH_2** Pip and $^+NCH_2$**CH_2**$(CH_2)_{11}CH_3$), 1.25-1.20 (m, 22H, $^+NCH_2CH_2$**$(CH_2)_{11}$**CH_3), 0.87 (t, 3H, CH_3); ^{13}CNMR (DMSO) δ ppm: 169.5, 139.9, 129.9, 129.7, 127.7, 60.3, 47.4, 31.8, 29.5, 29.5, 29.4, 29.3, 29.2, 29.0, 26.3, 22.6, 21.4, 21.1, 19.7, 14.4

[C_1C_{14}Pip]$_3$[Trim]: viscous white solid; yield 82%; ^{1}HNMR (DMSO) δ ppm: 8.50 (s, 3H, Ar), 3.30-3.28 (m, 18H, $^+$N**CH_2** Pip, $^+$N**CH_2**$CH_2(CH_2)_{11}CH_3$), 2.97 (s, 9H, 3 x $^+$N**CH_3**), 1.78-1.47 (m, 24H, $^+$N**CH_2**CH_2-), 1.25-1.23 (m, 66H, 3 x $^+NCH_2CH_2$**$(CH_2)_{11}$**CH_3), 0.85 (t, 3H, CH_3);

[C_1C_{14}Pip][Cin]: white solid; yield 98%; ^{1}HNMR (DMSO) δ ppm: 7.49-7.47 (m, 2H, Ar), 7.35-7.26 (m, 3H, Ar), 7.13 (d, J= 16 Hz, 2H), 6.41 (d, J= J= 16 Hz, 2H), 3.30 – 3.25 (m, 6H, 2 x $^+$N**CH_2** Pip, $^+$N**CH_2**$CH_2(CH_2)_{11}CH_3$), 2.97 (s, 3H, $^+$N**CH_3**), 1.78-1.73 (m, 4H, 2 x **- CH_2** Pip), 1.64-1.62 (m, 2H, **- CH_2** Pip), 1.56-1.47 (m, 2H, $^+$N**CH_2**$CH_2(CH_2)_{11}CH_3$), 1.25-1.23 (m, 22H, $^+NCH_2CH_2$**$(CH_2)_{11}$**CH_3), 0.85 (t, 3H, CH_3); ^{13}CNMR (DMSO) δ ppm: 169.9, 137.2, 136.9, 129.1, 128.9, 128.7, 127.5, 60.3, 47.2, 31.8, 29.5, 29.5, 29.4, 29.3, 29.2, 29.0, 26.4, 22.6, 21.4, 21.2, 19.8, 14.4

[C_1C_{14}Pip]$_2$[Sub]: viscous white solid; yield 94%; ^{1}HNMR ($CDCl_3$) δ ppm: 3.70-3.65 (m, 4H, 2 x $^+$N**CH_2**$CH_2(CH_2)_{11}CH_3$), 3.55-3.48 (m, 8H, 4 x $^+$N**CH_2** Pip), 3.25 (s, 6H, $^+$N**CH_3**), 2.20 (t, 4H, -**CH_2**COOH), 1.90-1.56 (m, 20 H, 2 x $^+NCH_2$**CH_2**$(CH_2)_{11}CH_3$, 2 x $^+NCH_2$**$CH_2CH_2CH_2$** Pip, and 2 x **CH_2**CH_2COOH), 1.33-1.23 (m, 48H, 2 x $^+NCH_2CH_2$**$(CH_2)_{11}$**CH_3 and **$(CH_2)_2$**CH_2CH_2COOH), 0.86 (t, 3H, CH_3); ^{13}CNMR (DMSO) δ ppm: 175.9, 60.2, 37.0, 31.9, 29.5, 29.5, 29.5, 29.4, 29.3, 29.2, 29.0, 26.4, 26.3, 22.6, 21.4, 21.2, 19.8, 14.4

[C_1C_{14}Pip]$_2$[Adip]: viscous white solid; yield 86%; ^{1}HNMR ($CDCl_3$) δ ppm: 8.11 (s, 2H, OH), 3.69-3.67 (m, 4H, 2 x $^+$N**CH$_2$** Pip), 3.57-3.50 (m, 8H, 4 x $^+$N**CH$_2$**Pip); 3.36 (s, 6H, 2 x $^+$N**CH$_3$**); 2.29 (s, 4H, 2 x **CH$_2$**COOH ; 1.90-1.65 (m, 20H, 2 x $^+NCH_2$**CH$_2$**$(CH_2)_{11}CH_3$, 2 x $^+NCH_2$**CH$_2$CH$_2$CH$_2$** Pip and 2 x **CH$_2$**CH_2COOH); 1.35-1.24 (m, 44H, 2 x $^+NCH_2CH_2$**(CH$_2$)$_{11}$**CH_3), ; 0.86 (t, 3H, CH_3); ^{13}CNMR ($CDCl_3$) δ ppm: 60.9, 48.7, 31.9, 29.6, 29.5, 29.4, 29.3, 29.3, 26.4, 25.5, 22.7, 21.9, 20.7, 20.1, 14.

Procedure for the synthesis of [C_8C_2OHIm]Br salt

A solution of *N*-(2-hydroxyethyl)imidazole (500 mg, 4.46 mmol) in acetonitrile (2 mL) was treated with a stoichiometric amount of 1-bromooctane (4.46 mmol, 861 mg). The reaction mixture was heated under reflux at 70 °C and stirred for 24 h. After this time, the solvent was removed under reduced pressure, and the resulting oil was washed three times with ethyl acetate (3 × 5 mL).

[C_8C_2OHIm]Br: yellow oil; yield 89%; ^{1}HNMR ($CDCl_3$) δ ppm: 9.62 (s, 1H, CH Im), 7.69-7.68 (m, 1H, CH Im), 7.38-7.36 (m, 1H, CH Im), 4.50-4.48 (m, 2H, $^+NCH_2$**CH$_2$**OH), 4.26-4.22 (m, 2H, $^+$N**CH$_2$**$CH_2(CH_2)_5CH_3$), 3.95-3.92 (m, 2H, $^+$N**CH$_2$**CH_2OH), 1.89-1.86 (m, 2H, $^+NCH_2$**CH$_2$**$(CH_2)_5CH_3$), 1.30-1.21 (m, 10H, $^+NCH_2CH_2$**(CH$_2$)$_5$**CH_3), 0.83 (t, 3H, CH_3); ^{13}CNMR ($CDCl_3$) δ ppm: 136.3, 123.4, 121.7, 60.0, 52.2, 50.1, 31.7, 30.1, 29.0, 28.9, 26.3, 22.5, 14.0

Procedure for the synthesis of [BzC_8Im]Br salt

In a two-neck round bottomed flask, benzyl bromide (100 mg, 0.58 mmol) was solubilized in isopropanol (2 mL), heating at 85 °C. In a second flask, 1-octyl-imidazole (0.58 mmol, 104.6 mg) was solubilized in isopropanol (0.5 mL) and added dropwise to the benzyl bromide solution. The rection mixture was then refluxed at 85 °C and stirred for 18 h. The solvent was removed under reduced pressure and the residue washed three times with diethyl ether (3 x 5 mL).

[BzC_8Im]Br: orange oil; 80%; ^{1}HNMR (DMSO) δ ppm: 9.37 (s, 1H, CH Im), 7.83-7.82 (m, 2H, 2 x CH Im), 7.43-7.39 (m, 5H, Ar), 5.43 (s, 2H, -$^+$N**CH$_2$**Bz), 4.19-4.15 (m, 2H, -$^+$N**CH$_2$** CH_2 $(CH_2)_5CH_3$),

1.80-1.76 (m, 2H, -$^+$NCH$_2$ **CH$_2$** (CH$_2$)$_5$CH$_3$), 1.28-1.18 (m, 10H, -$^+$NCH$_2$ CH$_2$ **(CH$_2$)$_5$**CH$_3$), 0.84 (t, 3H, CH$_3$); ^{13}CNMR (CDCl$_3$) δ ppm: 136.6, 133.2, 129.3, 129.0, 122.2, 122.1, 121.0, 53.1, 50.2, 31.5, 30.2, 28.9, 28.8, 26.2, 22.5, 14.0

Procedure for the synthesis of dicationic salts [*o*-xyl(C$_8$Im)$_2$]Br$_2$ and [*o*-xyl(C$_8$C$_1$C$_1$N)$_2$]Br$_2$

In a two-neck round bottomed flask, *α,α'*-dibromo-*o*-xylene (300 mg, 1.14 mmol) was dissolved in (3 mL) of isopropanol heating at 85 °C. In a second flask, 1-octyl-imidazole (2.2 eq, 4.16 mmol) or *N,N*-dimethyloctylamine (2.2 eq, 2.5 mmol) were dissolved in isopropanol (1.9 mL) and the solution was added, dropwise, to the α,α'-dibromo-xylene solution.

The reaction mixture was heated at 85 °C for 24 h and monitored by TLC. The solvent was removed under reduced pressure and the residue washed three times with diethyl ether (3 x 10 mL).

[*o*-xyl(C$_8$Im)$_2$]Br$_2$: white solid; yield 89%; ^{1}HNMR (CDCl$_3$) δ ppm: 10.03 (s, 2H, CH Im), 7.83-7.82 (m, 2H, CH Im), 7.39-7.37 (m, 4H, CH Im and Ar), 7.25-7.23 (m, 2H, Ar), 6.09 (s, 4H, **-CH$_2$**Bz), 4.29-4.25 (m, 4H, 2 x -$^+$**NCH$_2$** CH$_2$ (CH$_2$)$_5$CH$_3$), 1.90-1.84 (m, 4H, 2 x -$^+$NCH$_2$**CH$_2$** (CH$_2$)$_5$CH$_3$), 1.32-1.23 (m, 20H, 2 x -$^+$NCH$_2$CH$_2$**(CH$_2$)$_5$**CH$_3$), 0.85 (t, 6H, CH$_3$); ^{13}CNMR (CDCl$_3$) δ ppm: 136.5, 132.3, 130.2, 129.9, 123.3, 122.1, 50.5, 31.6, 30.1, 28.9, 28.9, 26.3, 22.6, 14.0.

[*o*-xyl(C$_8$C$_1$C$_1$)$_2$]Br$_2$: white solid; yield 93%; ^{1}HNMR (CDCl$_3$) δ ppm: 8.12-8.09 (m, 2H, CH Ar), 7.66-7.64 (m, 2H, CH Ar), 5.48 (s, 4H, **CH$_2$**Bz), 3.83-3.79 (m, 4H, 2 x -$^+$**NCH$_2$**CH$_2$(CH$_2$)$_5$CH$_3$), 3.25 (s, 12H, 4 x $^+$NCH$_3$), 1.74-1.70 (m, 4H, 2 x -$^+$NCH$_2$**CH$_2$**(CH$_2$)$_5$CH$_3$), 1.34-1.22 (m, 20H, 2 x -$^+$NCH$_2$CH$_2$**(CH$_2$)$_5$**CH$_3$), 0.84 (t, 6H, CH$_3$); ^{13}CNMR (DMSO) δ ppm: 135.9, 131.1, 130.1, 64.1, 63.4, 48.8, 31.6, 29.0, 28.9, 26.3, 22.5, 22.4, 14.4.

Procedure for the synthesis of dicationic salts [*o*-xyl(C$_{12}$Im)$_2$]Br$_2$

The salt [o-xyl(C$_{12}$Im)$_2$]Br$_2$ was synthetized through a two-step reaction process. In the first reaction step, 1-H-imidazole (200 mg, 3 mmol) was reacted with potassium hydroxide (KOH, 329 mg, 6 mmol) in acetonitrile (37 mL) at room temperature for 2 hours. Subsequently, 1-bromododecane

(0.712 mL, 3 mmol) was added dropwise to the reaction mixture, which was then stirred at room temperature for an additional 18 hours. The solvent was evaporated under vacuum and the residue was treated with water and extracted with dichloromethane until the aqueous phase reached a neutral pH. The organic layer was dried over anhydrous sodium sulfate (Na_2SO_4) and concentrated under vacuum, yielding 1-dodecyl-1H-imidazole.

In a second reaction step, *α,α'*-dibromo-*o*-xylene (268 mg, 1.01 mmol) was dissolved in (3 mL) of isopropanol heating at 85 °C. In a separate flask, the previously synthesized 1-dodecyl-1H-imidazole (530 mg, 2.24 mmol) was dissolved in 2 mL of isopropanol and then added dropwise to the *α,α'*-dibromo-*o*-xylene solution.

The resulting mixture was heated at 85 °C for 24 hours, and the progress of the reaction was monitored by TLC. Upon completion, the solvent was removed under reduced pressure, and the crude product was washed three times with acetone (3 × 10 mL) followed by diethyl ether (3 × 10 mL).

[*o*-xyl(C_{12}Im)$_2$]Br_2: white solid; yield 95%; ^{1}HNMR ($CDCl_3$) δ ppm: 10.04 (s, 2H, CH Im), 7.84-7.83 (m, 2H, CH Im), 7.40-7.39 (m, 2H, CH Im), 7.35-7.32 (m, 2H, CH Ar), 7.24-7.22 (m, 2H, CH Ar), 6.06 (s, 4H, -**CH_2**Bz), 4.27-4.23 (m, 4H, 2 x -$^+$**NCH_2** CH_2 $(CH_2)_9CH_3$), 1.87-1.83 (m, 4H, 2 x -$^+$NCH_2**CH_2** $(CH_2)_9CH_3$), 1.30-1.21 (m, 18H, 2 x -$^+$NCH_2CH_2**$(CH_2)_9$**CH_3), 0.84 (t, 3H, CH_3); ^{13}CNMR ($CDCl_3$) δ ppm: 136.5, 132.3, 130.2, 129.9, 123.2, 122.0, 50.8, 50.5, 31.8, 30.1,.29.6, 29.5, 29.4, 29.3, 28.8, 26.3, 22.7, 14.1.

Procedure for the synthesis of dicationic salt [*o*-xyl(C_8Im)$_2$][Docu]$_2$

[*o*-xyl(C8Im)$_2$]Br_2 (250 mg, 0.40 mmol) was dissolved in dichloromethane (3 mL) under stirring. A stoichiometric amount of dioctyl sodium sulfosuccinate salt (AOT) (2.0 equiv, 0.80 mmol, 356 mg) was added slowly to the solution while maintaining the reaction flask in an ice bath. The mixture was then stirred at room temperature for 72 h. After completion, the resulting precipitate of NaBr was removed by filtration. The organic phase was washed three times with an equal volume of brine and twice with deionized water. To confirm complete removal of bromide ions, each aqueous layer was

tested with a silver nitrate ($AgNO_3$) solution. The absence of precipitate in the final wash indicated successful elimination of residual bromide. The organic layer was dried over anhydrous sodium sulfate (Na_2SO_4), filtered, and the solvent was removed under reduced pressure to afford the final product as a residue.

[*o*-xyl(C_8Im)$_2$][Docu]$_2$: transparent oil; yield 89%; ^{1}HNMR ($CDCl_3$) δ ppm: 9.40 (s, 2H, CH Im), 7.61 (t, 2H, CH Im), 7.41-7.38 (m, 2H, CH Ar), 7.27 (t, 2H, CH Im), 7.15-7.13 (m, 2H, CH, Ar), 5.70 (s, 4H, -**CH_2**Bz), 4.22 (t, 4H, 2 x -$^+$N**CH_2** CH_2 $(CH_2)_5CH_3$), 4.03-3.88 (m, 10H, 4 x -**CH_2**OCO and 2 x **CH**SO_3^-), 3.16-2.99 (m, 4H, 2 x -**CH_2**CO), 1.85 (t, 4H, 2 x -$^+$NCH_2 **CH_2**$(CH_2)_5CH_3$), 1.61-1.50 (m, 4H, 4 x **CH**CH_2CH_3), 1.33-1.22 (m, 54H), 0.89-0.81 (m, 30H, CH_3); ^{13}C NMR ($CDCl_3$) δ 171.5, 169.1, 137.0, 132.3, 130.0, 129.7, 123.3, 122.1, 67.7, 67.6, 67.1, 61.9, 50.4, 50.2, 38.6, 38.5, 38.4, 34.0, 31.6, 30.3, 30.2, 30.1, 30.0, 29.9, 29.0, 28.9, 28.8, 28.8, 26.3, 23.6, 23.4, 22.9, 22.6, 14.1, 14.0, 10.9, 10.8, 10.8, 10.7, 0.9.

Procedure for the synthesis of dicationic salts [*o*-xyl(C_8Im)$_2$][Tos]$_2$ and [*o*-xyl($C_8C_1C_1$N)$_2$] [Tos]$_2$

As previously reported, anion exchange using Amberlite IRA-400 resin (chloride form) enabled the conversion of bromide salts **[*o*-xyl(C_8Im)$_2$]Br_2** and **[*o*-xyl($C_8C_1C_1$)$_2$]Br_2** into their corresponding tosylate derivatives.[83]

A defined amount of Amberlite IRA-400 resin (3.28 g per mmol of bromide salt) was packed into a column using distilled water. To convert the resin from its chloride to the hydroxide form, it was first washed with an aqueous NaOH solution (10% w/v in bi-distilled water), prepared by dissolving 52 mg of NaOH per gram of Amberlite IRA-400 resin. Washing was continued until the eluate reached a neutral pH.

Subsequently, the bromide salt **[*o*-xyl(C_8Im)$_2$]Br_2** (500 mg, 0.8 mmol) or **[*o*-xyl($C_8C_1C_1$)$_2$]Br_2** (250 mg, 0.43 mmol) was dissolved in a methanol/water binary mixture (70:30, v/v) and loaded onto the column, using the same solvent mixture as the eluent. The eluate was collected, until a neutral pH was observed at the column outlet, in a flask containing a stoichiometric amount of *p*-Toluenesulfonic

acid monohydrate (2 eq) The resulting solution was stirred at room temperature overnight and then concentrated under reduced pressure. The residue was washed with diethyl ether (3 × 10 mL), and the crystallized product was isolated and dried under vacuum.

[*o*-xyl(C_8Im)$_2$][Tos]$_2$: pink solid; yield 88%; ^{1}HNMR ($CDCl_3$) δ ppm: 9.74 (s, 2H, CH Im), 7.68-7.64 (m, 6H, 2 x CH Im and 4 x CH Ar), 7.40-7.37 (m, 2H, CH Im), 7.31 (s, 2H, CH Im), 7.23-7.20 (m, 2H, CH Ar), 7.12 (d, J= 12 Hz, 4H, Ar), 5.86 (s, 4H, -**CH_2**Bz), 4.13 (t, 4H, 2 x -$^+$N**CH_2** CH_2 $(CH_2)_5CH_3$), 2.34 (s, 6H, CH_3 Tos), 1.77 (t, 4H, 2 x -$^+$NCH_2 **CH_2**$(CH_2)_5CH_3$), 1.27-1.22 (m, 20H, 2 x -$^+$NCH_2 CH_2**$(CH_2)_5$**CH_3), 0.88 (t, 6H, CH_3); ^{13}C NMR ($CDCl_3$) δ 143.2, 139.6, 137.1, 132.4, 130.2, 130.0, 128.8, 125.8, 123.1, 122.2, 50.4, 50.1, 31.8, 30.1, 29.1, 28.9, 26.2, 22.5, 21.4, 14.00.

[*o*-xyl($C_8C_1C_1$)$_2$] [Tos]$_2$: aspetto: yield 50 %: ^{1}HNMR ($CDCl_3$) δ ppm: 7.85 (s, 2H, CH Ar), 7.71 (d, J= 8 Hz, CH Ar), 7.56 (s, 2H, CH Ar), 7.13 (d, J= 8 Hz, CH Ar), 5.14 (s, 4H, -**CH_2**Bz), 3.03 (s, 12H, 4 x $^+NCH_3$), 3.48 (s, 4H, 2 x -$^+$N**CH_2** CH_2 $(CH_2)_5CH_3$), 2.33 (s, 6H, CH_3 Tos), 1.59 (s, 4H, 2 x -$^+$NCH_2 **CH_2**$(CH_2)_5CH_3$), 1.25-1.12 (m, 20H, 2 x -$^+$NCH_2 CH_2**$(CH_2)_5$**CH_3), 0.87 (t, 6H, CH_3);

Figure S1. Thermogravimetric (TGA) and derivative thermogravimetric curves (DTG) of synthetized salts

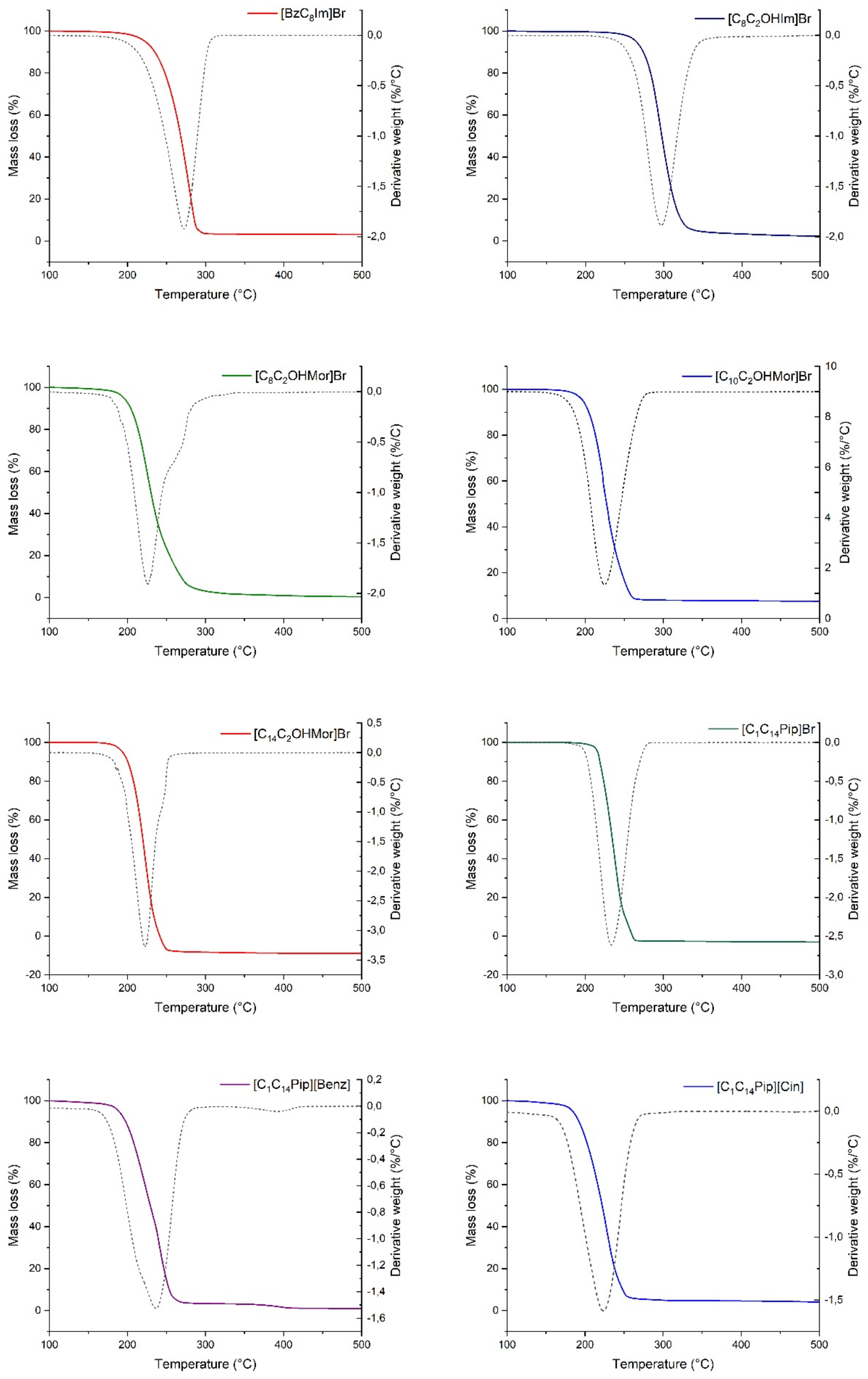

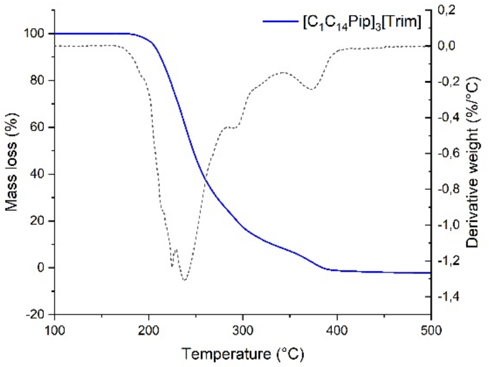
[C$_1$C$_{14}$Pip]$_3$[Trim]
Mass loss (%)
Derivative weight (%/°C)
Temperature (°C)

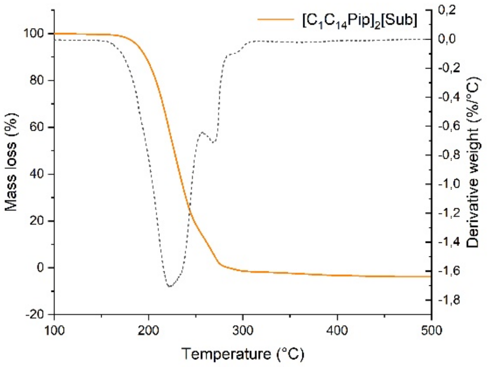
[C$_1$C$_{14}$Pip]$_2$[Sub]
Mass loss (%)
Derivative weight (%/°C)
Temperature (°C)

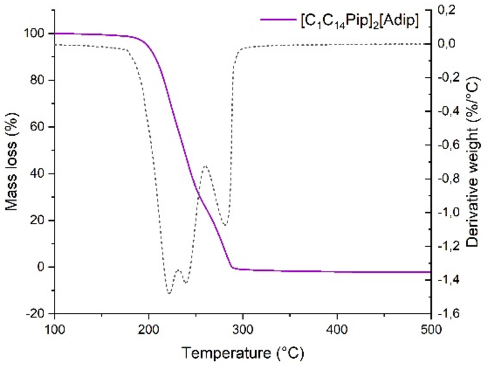
[C$_1$C$_{14}$Pip]$_2$[Adip]
Mass loss (%)
Derivative weight (%/°C)
Temperature (°C)

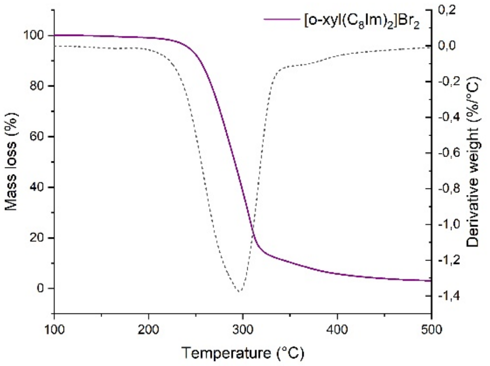
[o-xyl(C$_8$Im)$_2$]Br$_2$
Mass loss (%)
Derivative weight (%/°C)
Temperature (°C)

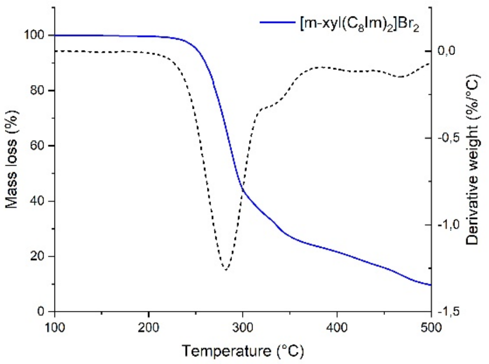
[m-xyl(C$_8$Im)$_2$]Br$_2$
Mass loss (%)
Derivative weight (%/°C)
Temperature (°C)

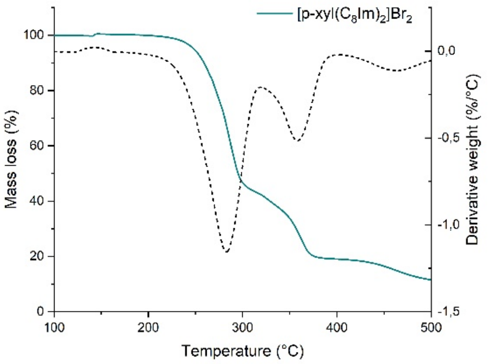
[p-xyl(C$_8$Im)$_2$]Br$_2$
Mass loss (%)
Derivative weight (%/°C)
Temperature (°C)

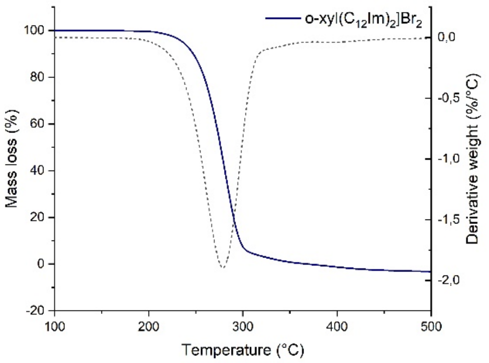
o-xyl(C$_{12}$Im)$_2$]Br$_2$
Mass loss (%)
Derivative weight (%/°C)
Temperature (°C)

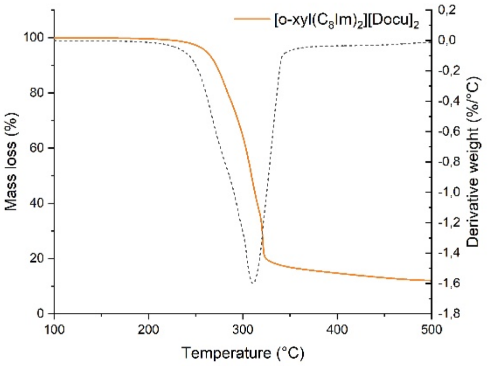
[o-xyl(C$_8$Im)$_2$][Docu]$_2$
Mass loss (%)
Derivative weight (%/°C)
Temperature (°C)

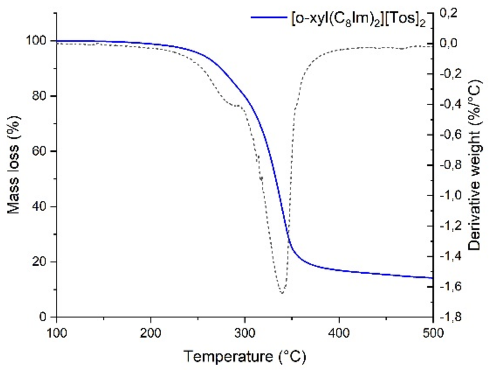
[o-xyl(C8Im)2][Tos]2
Mass loss (%)
Derivative weight (%/°C)
Temperature (°C)

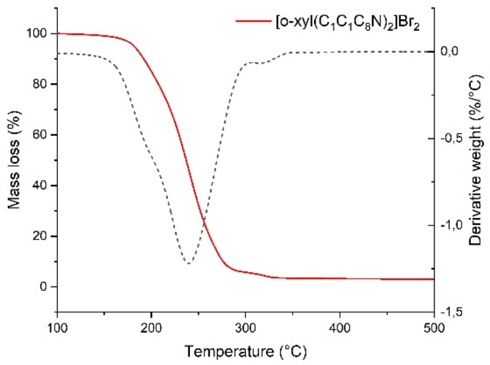
[o-xyl(C1C1C8N)2]Br2
Mass loss (%)
Derivative weight (%/°C)
Temperature (°C)

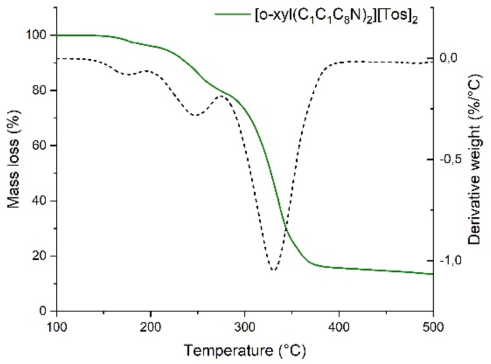
[o-xyl(C1C1C8N)2][Tos]2
Mass loss (%)
Derivative weight (%/°C)
Temperature (°C)

Table S1. Thermal parameters of synthetized salts obtained from TGA and DTG profiles

**Entry**	**Sample name**	**T_{peak} (°C)**	**T_d****	**T_{onset}**
**1**	[BzC_8Im]Br	272.07	222.59	248.18
**2**	[C_8C_2OHIm]Br	296.78	264.77	276.61
**3**	[C_4C_2OHMor]Br	240.73	204.63	215.53
**4**	[C_8C_2OHMor]Br	225.74	195.38	205.22
**5**	[$C_{10}C_2OHMor$]Br	224.91	197.18	207.02
**6**	[$C_{14}C_2OHMor$]Br	222.83	193.64	204.29
**7**	[$C_1C_{14}Pip$]Br	233.55	215.94	219.07
**8**	[$C_1C_{14}Pip$][Benz]	236.82*	188.85	207.24
**9**	[$C_1C_{14}Pip$][Cin]	223.41	184.04	198.02
**10**	[$C_1C_{14}Pip$]$_3$[Trim]	238.11*	205.53	207.91
**11**	[$C_1C_{14}Pip$]$_2$[Sub]	221.94*	188.26	197.99
**12**	[$C_1C_{14}Pip$]$_2$[Adip]	221.93*	198.24	202.96
**13**	[*o*-xyl(C_8Im)$_2$]Br_2	296.46	242.95	258.7
**14**	[*m*-xyl(C_8Im)$_2$]Br_2	282.12	250.73	258.09
**15**	[*p*-xyl(C_8Im)$_2$]Br_2	282.92	248.46	260.34
**16**	[*o*-xyl($C_{12}Im$)$_2$]Br_2	278.87	236.21	254.55
**17**	[*o*-xyl(C_8Im)$_2$][Docu]$_2$	320.88	261.24	297.28
**18**	[*o*-xyl(C_8Im)$_2$][Tos]$_2$	339.72	254.12	302.48
**19**	[*o*-xyl($C_1C_1C_8N$)$_2$]Br_2	239.69	181.64	201.90
**20**	[*o*-xyl($C_1C_1C_8N$)$_2$][Tos]$_2$	330.72	213.93	292.80

*T_{peak}: temperature of most intense peak of derivative thermogravimetric curve (DTG)
**T_d: temperature corresponding to the 5% weight loss

Figure S2. DSC thermograms of synthetized salts

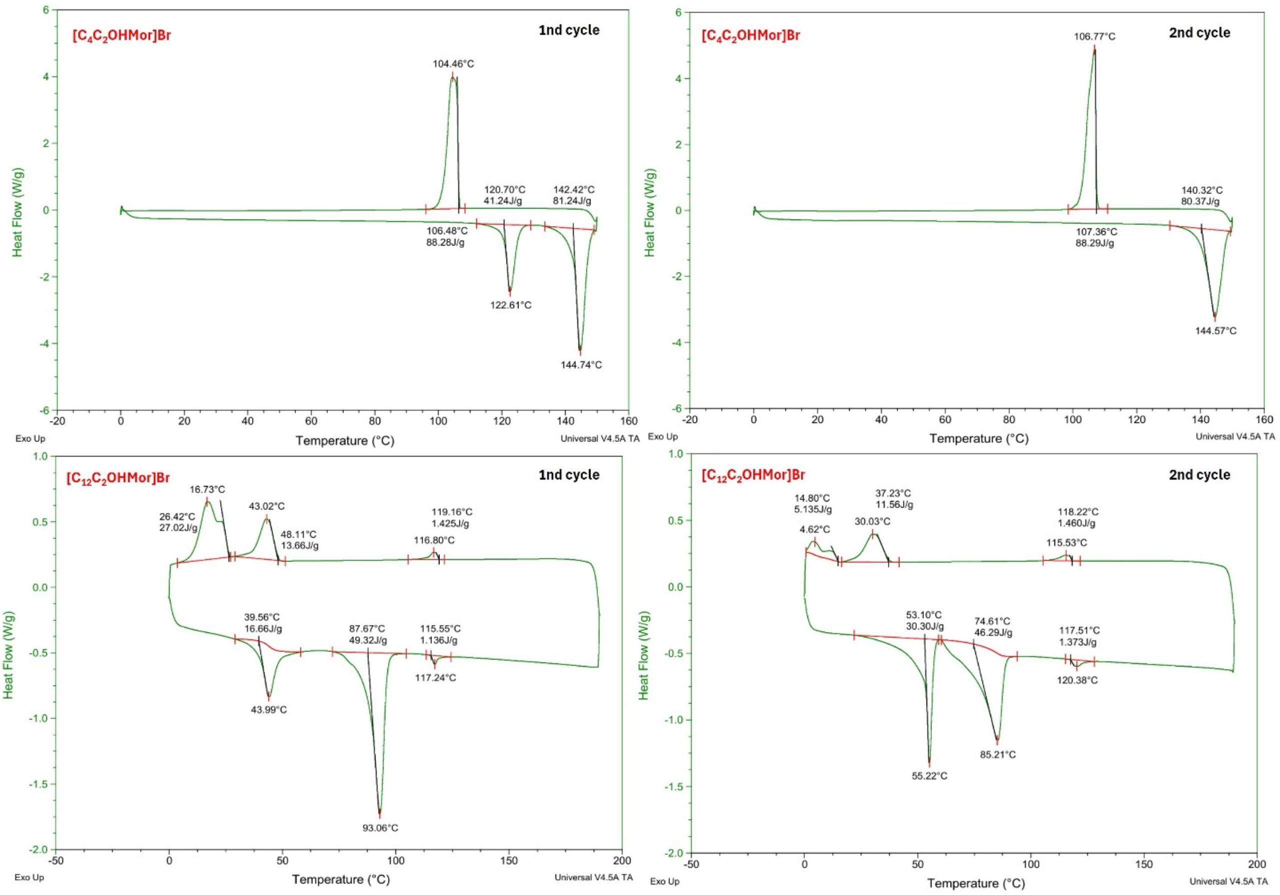

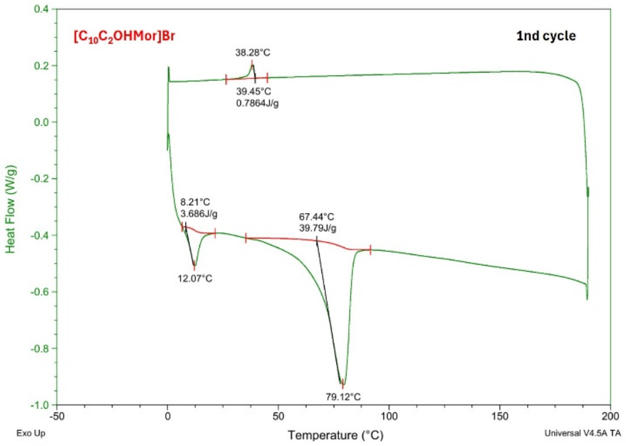
[C10C2OHMor]Br
1nd cycle
38.28°C
39.45°C
0.7864J/g
8.21°C
3.686J/g
12.07°C
67.44°C
39.79J/g
79.12°C
Heat Flow (W/g)
Temperature (°C)
Exo Up
Universal V4.5A TA

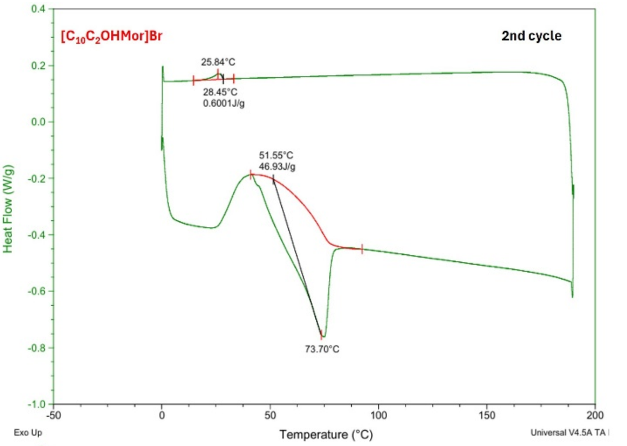
[C10C2OHMor]Br
2nd cycle
25.84°C
28.45°C
0.6001J/g
51.55°C
46.93J/g
73.70°C
Heat Flow (W/g)
Temperature (°C)
Exo Up

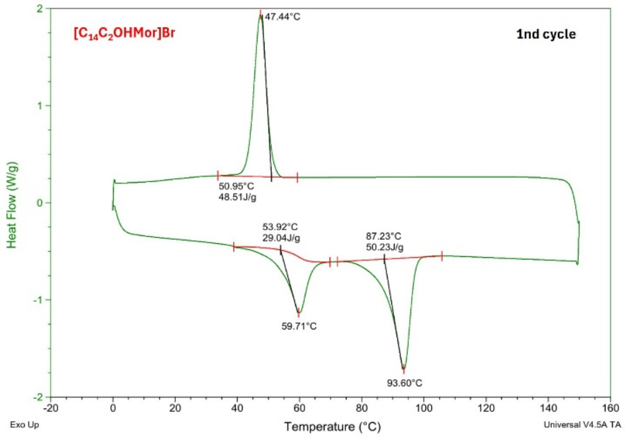
[C14C2OHMor]Br
1nd cycle
47.44°C
50.95°C
48.51J/g
53.92°C
29.04J/g
59.71°C
87.23°C
50.23J/g
93.60°C
Heat Flow (W/g)
Temperature (°C)
Exo Up
Universal V4.5A TA

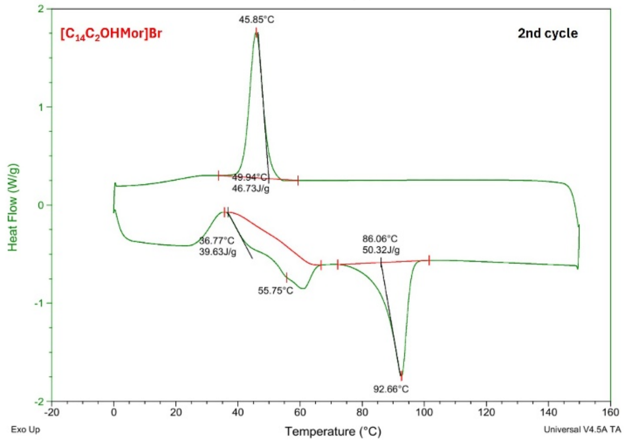
[C14C2OHMor]Br
2nd cycle
45.85°C
49.94°C
46.73J/g
36.77°C
39.63J/g
55.75°C
86.06°C
50.32J/g
92.66°C
Heat Flow (W/g)
Temperature (°C)
Exo Up
Universal V4.5A TA

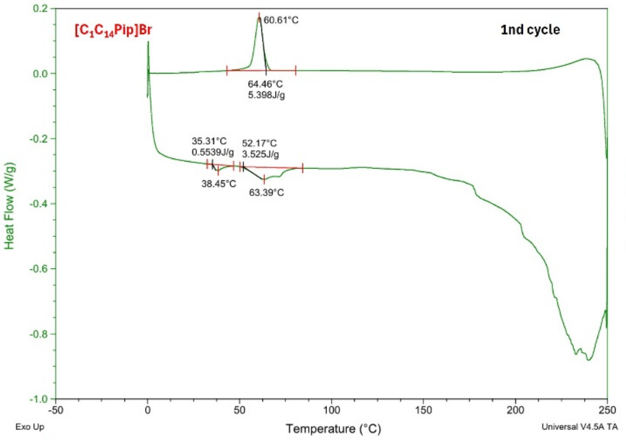
[C1C14Pip]Br
1nd cycle
60.61°C
64.46°C
5.398J/g
35.31°C
0.5539J/g
38.45°C
52.17°C
3.525J/g
63.39°C
Heat Flow (W/g)
Temperature (°C)
Exo Up
Universal V4.5A TA

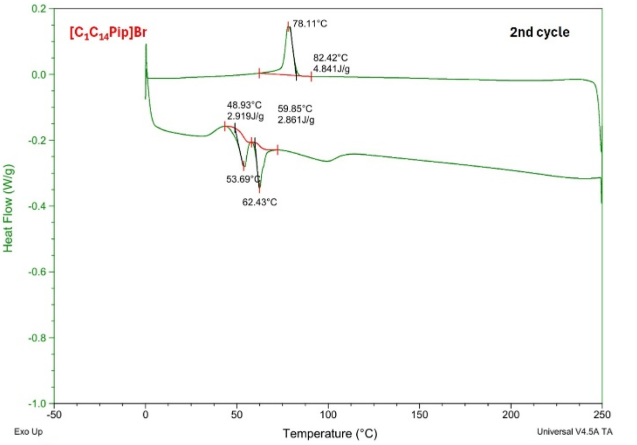
[C1C14Pip]Br
2nd cycle
78.11°C
82.42°C
4.841J/g
48.93°C
2.919J/g
53.69°C
59.85°C
2.861J/g
62.43°C
Heat Flow (W/g)
Temperature (°C)
Exo Up
Universal V4.5A TA

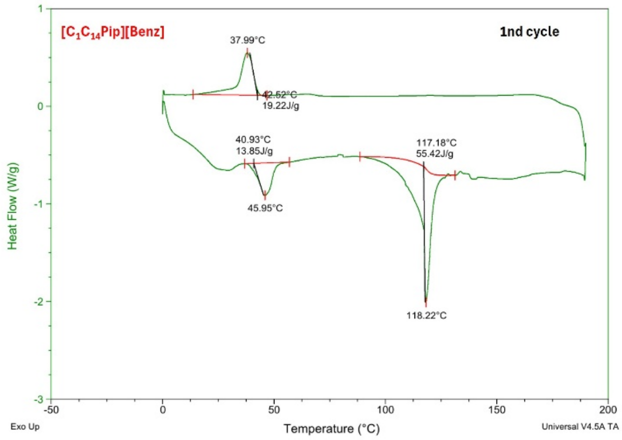
[C1C14Pip][Benz]
1nd cycle
37.99°C
42.52°C
19.22J/g
40.93°C
13.85J/g
45.95°C
117.18°C
55.42J/g
118.22°C
Heat Flow (W/g)
Temperature (°C)
Exo Up
Universal V4.5A TA

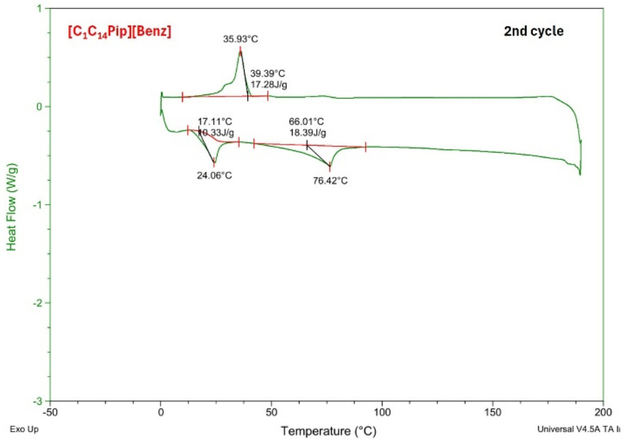
[C1C14Pip][Benz]
2nd cycle
35.93°C
39.39°C
17.28J/g
17.11°C
10.33J/g
24.06°C
66.01°C
18.39J/g
76.42°C
Heat Flow (W/g)
Temperature (°C)
Exo Up

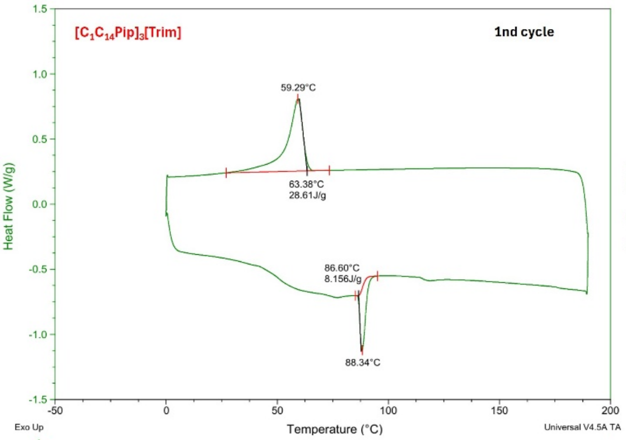
$[C_1C_{14}Pip]_3[Trim]$
1nd cycle
59.29°C
63.38°C
28.61J/g
86.60°C
8.156J/g
88.34°C
Heat Flow (W/g)
Temperature (°C)
Exo Up
Universal V4.5A TA

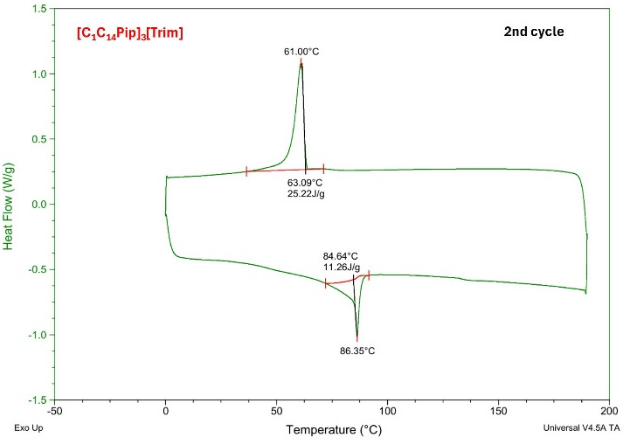
$[C_1C_{14}Pip]_3[Trim]$
2nd cycle
61.00°C
63.09°C
25.22J/g
84.64°C
11.26J/g
86.35°C
Heat Flow (W/g)
Temperature (°C)
Exo Up
Universal V4.5A TA

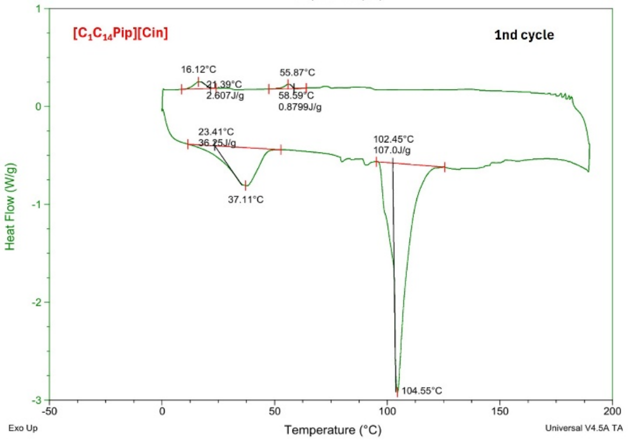
$[C_1C_{14}Pip][Cin]$
1nd cycle
16.12°C
21.39°C
2.607J/g
55.87°C
58.59°C
0.8799J/g
23.41°C
36.25J/g
37.11°C
102.45°C
107.0J/g
104.55°C
Heat Flow (W/g)
Temperature (°C)
Exo Up
Universal V4.5A TA

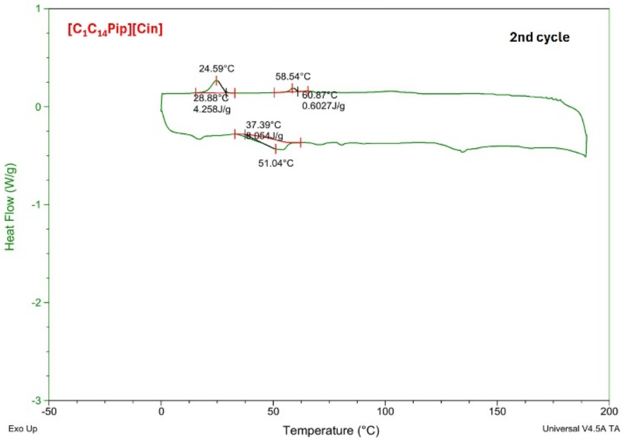
$[C_1C_{14}Pip][Cin]$
2nd cycle
24.59°C
28.88°C
4.258J/g
58.54°C
60.87°C
0.6027J/g
37.39°C
8.954J/g
51.04°C
Heat Flow (W/g)
Temperature (°C)
Exo Up
Universal V4.5A TA

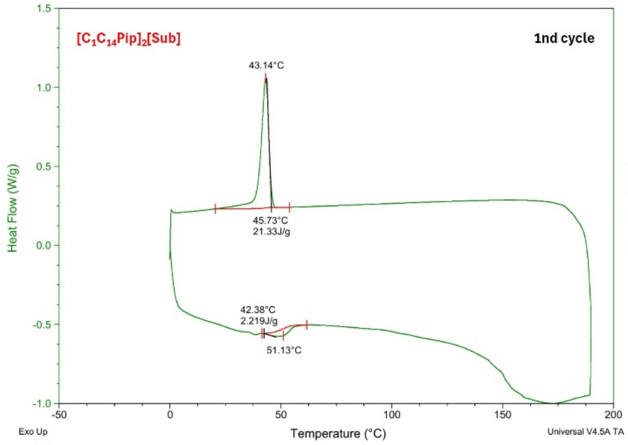
$[C_1C_{14}Pip]_2[Sub]$
1nd cycle
43.14°C
45.73°C
21.33J/g
42.38°C
2.219J/g
51.13°C
Heat Flow (W/g)
Temperature (°C)
Exo Up
Universal V4.5A TA

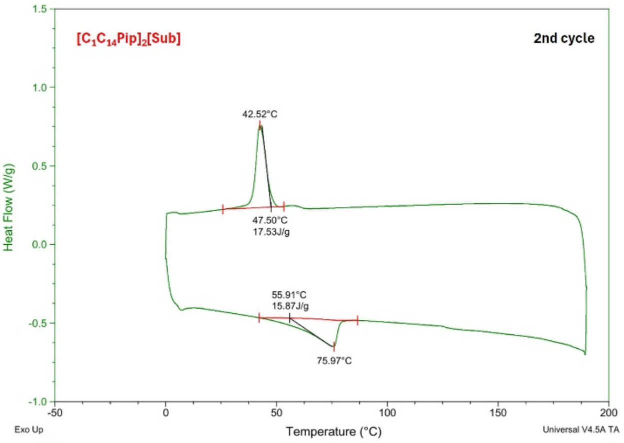
$[C_1C_{14}Pip]_2[Sub]$
2nd cycle
42.52°C
47.50°C
17.53J/g
55.91°C
15.87J/g
75.97°C
Heat Flow (W/g)
Temperature (°C)
Exo Up
Universal V4.5A TA

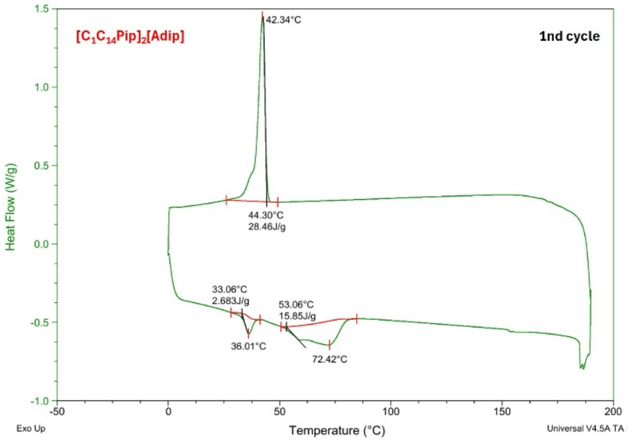
$[C_1C_{14}Pip]_2[Adip]$
1nd cycle
42.34°C
44.30°C
28.46J/g
33.06°C
2.683J/g
36.01°C
53.06°C
15.85J/g
72.42°C
Heat Flow (W/g)
Temperature (°C)
Exo Up
Universal V4.5A TA

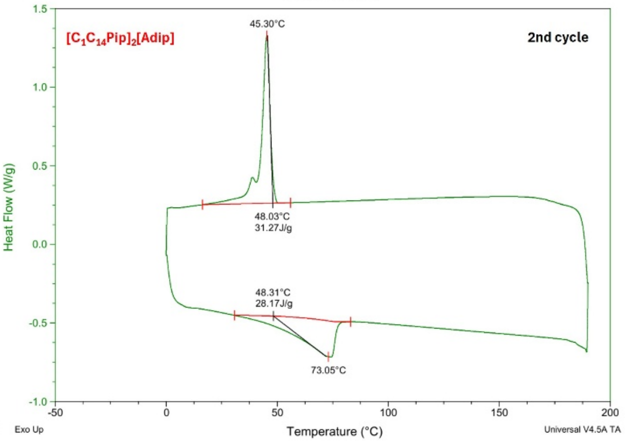
$[C_1C_{14}Pip]_2[Adip]$
2nd cycle
45.30°C
48.03°C
31.27J/g
48.31°C
28.17J/g
73.05°C
Heat Flow (W/g)
Temperature (°C)
Exo Up
Universal V4.5A TA

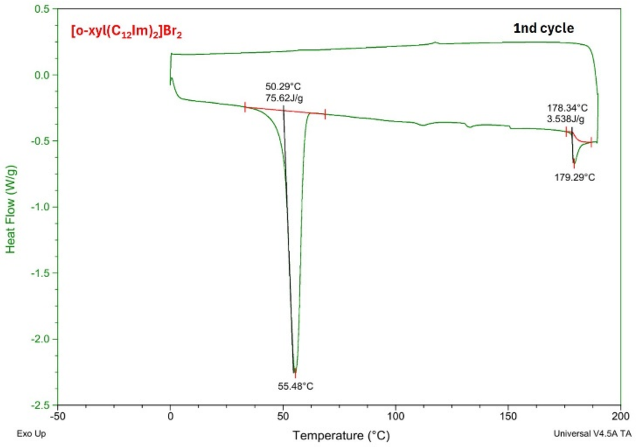
[o-xyl(C12Im)2]Br2
1nd cycle
50.29°C
75.62J/g
55.48°C
178.34°C
3.538J/g
179.29°C
Heat Flow (W/g)
Temperature (°C)
Exo Up
Universal V4.5A TA

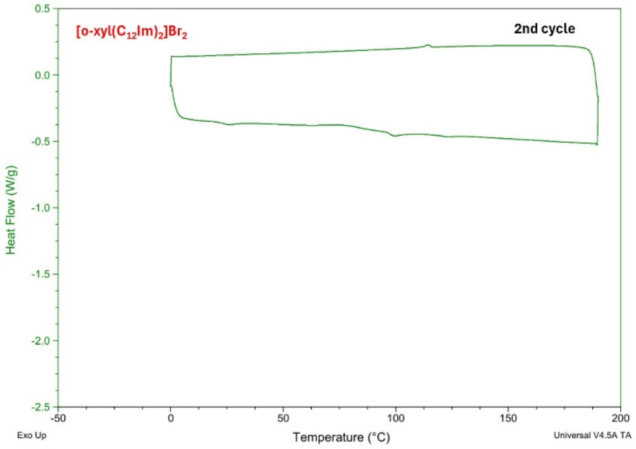
[o-xyl(C12Im)2]Br2
2nd cycle
Heat Flow (W/g)
Temperature (°C)
Exo Up
Universal V4.5A TA

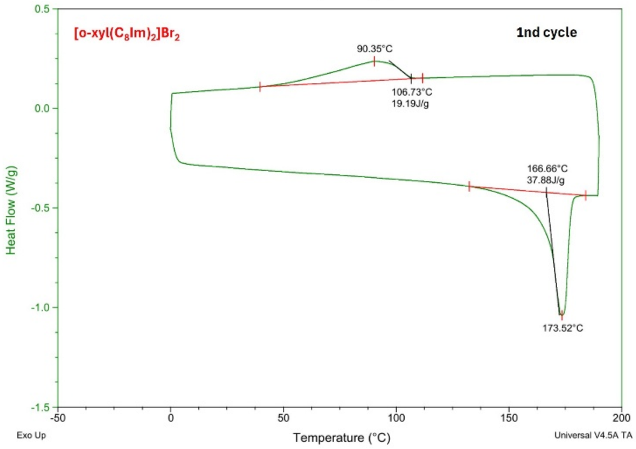
[o-xyl(C8Im)2]Br2
1nd cycle
90.35°C
106.73°C
19.19J/g
166.66°C
37.88J/g
173.52°C
Heat Flow (W/g)
Temperature (°C)
Exo Up
Universal V4.5A TA

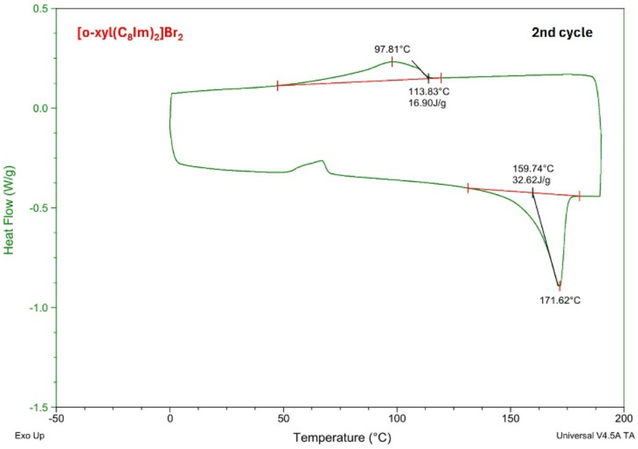
[o-xyl(C8Im)2]Br2
2nd cycle
97.81°C
113.83°C
16.90J/g
159.74°C
32.62J/g
171.62°C
Heat Flow (W/g)
Temperature (°C)
Exo Up
Universal V4.5A TA

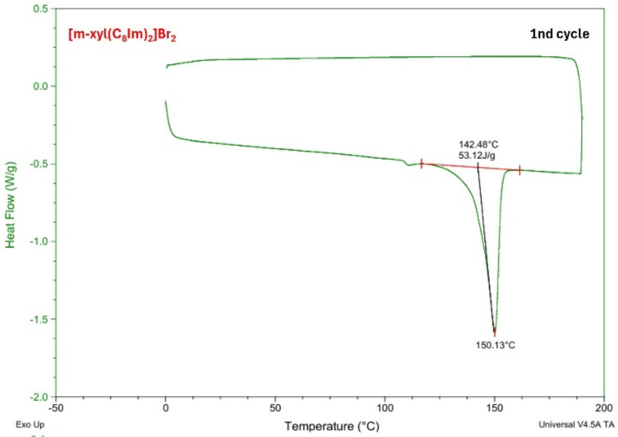
[m-xyl(C8Im)2]Br2
1nd cycle
142.48°C
53.12J/g
150.13°C
Heat Flow (W/g)
Temperature (°C)
Exo Up
Universal V4.5A TA

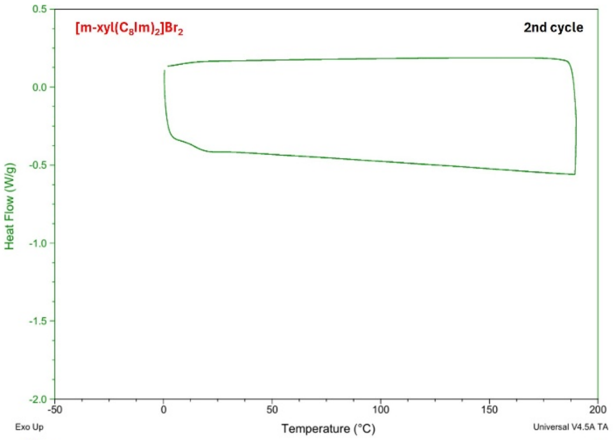
[m-xyl(C8Im)2]Br2
2nd cycle
Heat Flow (W/g)
Temperature (°C)
Exo Up
Universal V4.5A TA

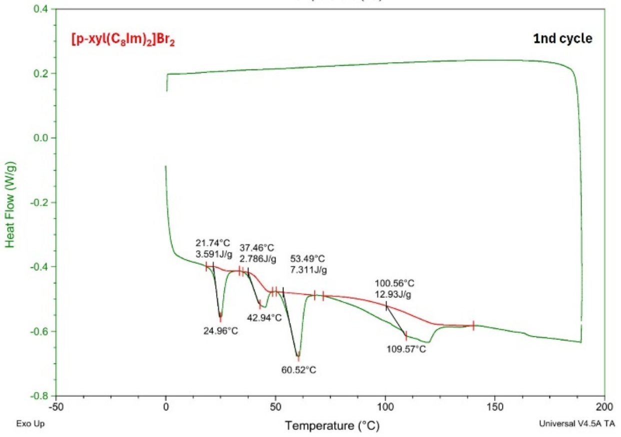
[p-xyl(C8Im)2]Br2
1nd cycle
21.74°C
3.591J/g
37.46°C
2.786J/g
53.49°C
7.311J/g
100.56°C
12.93J/g
24.96°C
42.94°C
60.52°C
109.57°C
Heat Flow (W/g)
Temperature (°C)
Exo Up
Universal V4.5A TA

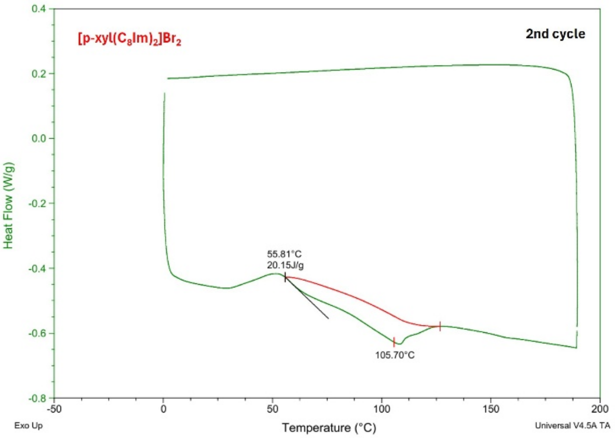
[p-xyl(C8Im)2]Br2
2nd cycle
55.81°C
20.15J/g
105.70°C
Heat Flow (W/g)
Temperature (°C)
Exo Up
Universal V4.5A TA

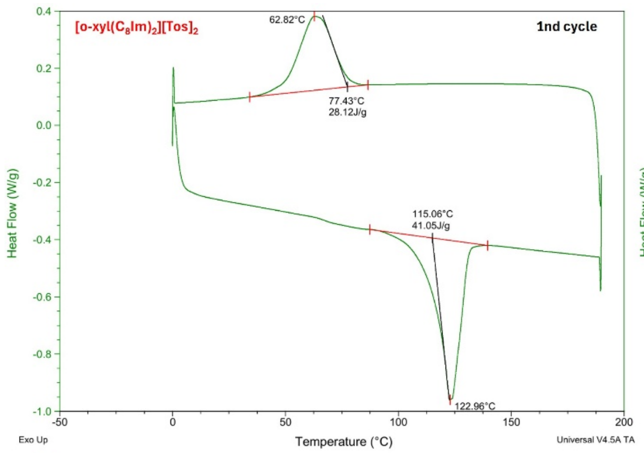
[o-xyl(C8Im)2][Tos]2
1nd cycle
62.82°C
77.43°C
28.12J/g
115.06°C
41.05J/g
122.96°C
Heat Flow (W/g)
Temperature (°C)
Exo Up
Universal V4.5A TA

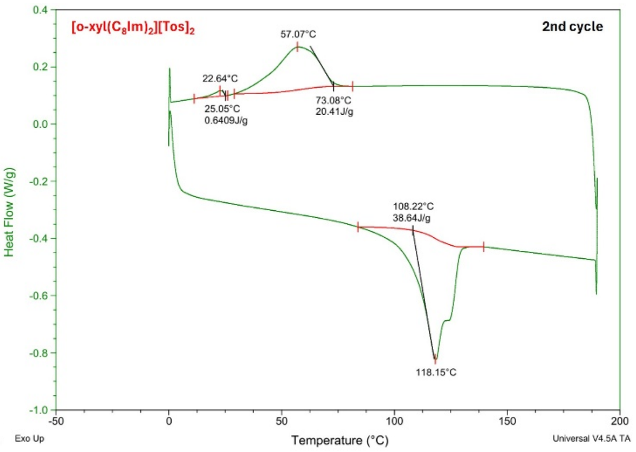
[o-xyl(C8Im)2][Tos]2
2nd cycle
57.07°C
22.64°C
25.05°C
0.6409J/g
73.08°C
20.41J/g
108.22°C
38.64J/g
118.15°C
Heat Flow (W/g)
Temperature (°C)
Exo Up
Universal V4.5A TA

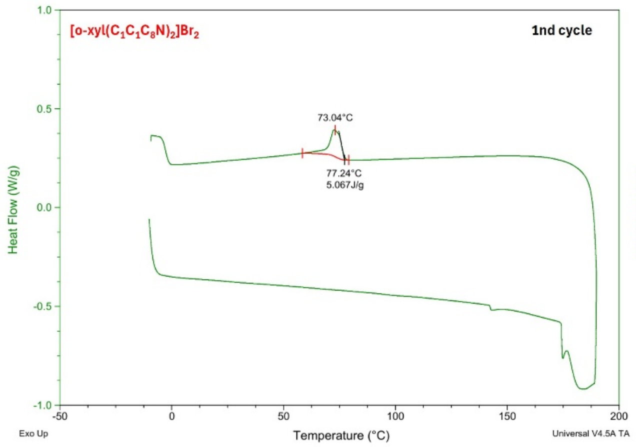
[o-xyl(C1C1C8N)2]Br2
1nd cycle
73.04°C
77.24°C
5.067J/g
Heat Flow (W/g)
Temperature (°C)
Exo Up
Universal V4.5A TA

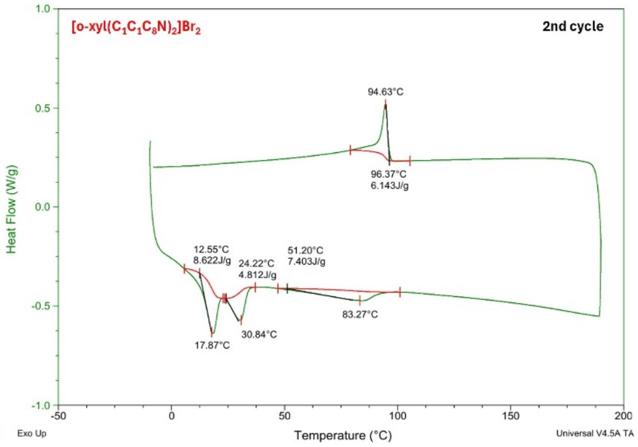
[o-xyl(C1C1C8N)2]Br2
2nd cycle
94.63°C
96.37°C
6.143J/g
12.55°C
8.622J/g
24.22°C
4.812J/g
51.20°C
7.403J/g
17.87°C
30.84°C
83.27°C
Heat Flow (W/g)
Temperature (°C)
Exo Up
Universal V4.5A TA

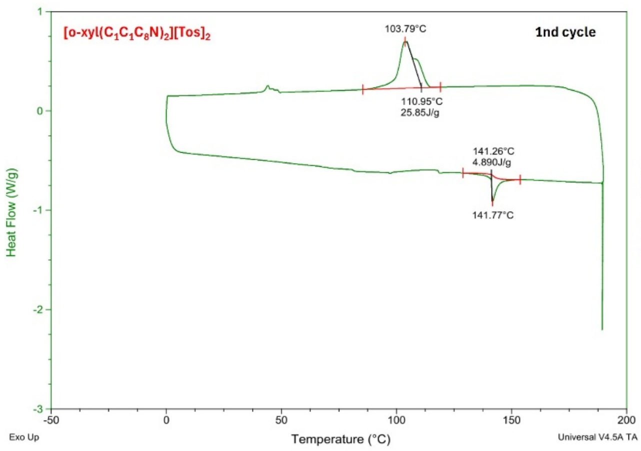
[o-xyl(C1C1C8N)2][Tos]2
1nd cycle
103.79°C
110.95°C
25.85J/g
141.26°C
4.890J/g
141.77°C
Heat Flow (W/g)
Temperature (°C)
Exo Up
Universal V4.5A TA

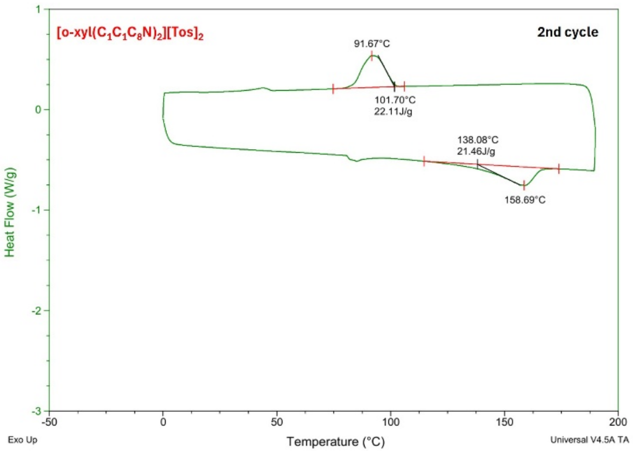
[o-xyl(C1C1C8N)2][Tos]2
2nd cycle
91.67°C
101.70°C
22.11J/g
138.08°C
21.46J/g
158.69°C
Heat Flow (W/g)
Temperature (°C)
Exo Up
Universal V4.5A TA
